# Assessing Second-Order Perturbative Corrections to Restricted Active Space CI for Valence Excitations in Organic Molecules

**DOI:** 10.1021/acs.jpca.5c06818

**Published:** 2025-12-06

**Authors:** Janaarthana Babu Perumal Marisami, David Casanova

**Affiliations:** † 226245 Donostia International Physics Center (DIPC), 20018 Donostia, Euskadi, Spain; ‡ Polimero eta Material Aurreratuak: Fisika, Kimika eta Teknologia Saila, Euskal Herriko Unibertsitatea (UPV/EHU), PK 1072, 20080 Donostia, Euskadi, Spain; § IKERBASQUE−Basque Foundation for Science, Bilbao, Euskadi 48009, Spain

## Abstract

We benchmark second-order perturbative corrections to the Restricted Active Space Configuration Interaction in the hole and particle approximation, RAS­(*h*,*p*), for valence singlet and triplet excitations in a set of organic molecules. Two partitioning schemes, Epstein–Nesbet (EN) and Davidson–Kapuy (DK), were assessed against NEVPT2 and reference data from the literature. The lack of dynamic correlation in RAS­(*h*,*p*) leads to a systematic overestimation of singlet excitation energies by ∼0.8 eV, with much smaller errors for triplets. EN perturbation provides only marginal improvement and increases statistical scatter, whereas DK yields a more consistent correction but tends to overcompensate. Introducing an energy level shift in the DK partition effectively removes this bias, producing excitation energies of comparable accuracy to NEVPT2. An optimal shift of ε ≈ 0.55 a.u. for singlets and slightly larger values for triplets was found to be broadly transferable across the data set, with ε in the range 0.4–0.6 a.u. offering a robust compromise. Analysis of second-order contributions shows that the dominant 1*h*1*p* terms act synergistically with the variational singles, resembling a state-specific orbital relaxation, while the more expensive 1*h*2*p* and 2*h*2*p* terms have a minor impact, suggesting a route to cheaper, targeted perturbative schemes. DK-based corrections exhibit weak basis-set dependence, enabling efficient composite strategies in which large-basis RAS­(*h*,*p*) energies are combined with small-basis DK+shift corrections, achieving significant computational savings with minimal loss of accuracy.

## Graphical abstract

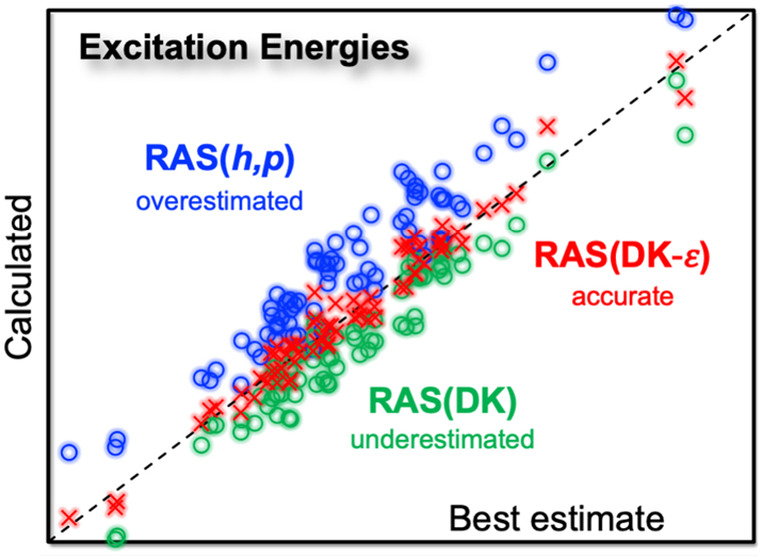

## 1 Introduction

Multiconfiguration (MC) wave function methods represent a rather balanced approach to describe extended regions of the molecular potential surface. In particular, MC methods are very efficient in the qualitative description of degenerate systems. These methods are based on the representation of the electronic wave function in terms of more than one Slater determinant. Some of the most prominent examples among the plethora of MC theories are the MC self-consistent field (MCSCF),^1^ the complete active space self-consistent field (CASSCF),^2,3^ generalized valence bond (GVB),^4–8^ or the antisymmetric product of strongly orthogonal geminals (APSG).^9–11^ But, in spite of their ability to describe various and challenging molecular electronic structures, MC theories are, in general, unable to capture the necessary amounts of dynamic correlation for the computation of relative energies with chemical accuracy. Commonly, the missed correlation can be added on top of a MC wave function using multireference configuration interaction (MRCI),^12,13^ multireference coupled cluster (MRCC),^14–16^ or multireference perturbation theory (MRPT)^17–19^ schemes. In MRPT there is not a unique or natural procedure to split the Hamiltonian in a zero-order part and a perturbation. This arbitrariness has given rise to the development of a large variety of MCPT approaches.^20–27^ In general, it is well accepted that the truncation of the perturbation expansion to the second-order energy correction produces accurate enough results for predictive chemistry.

From a different perspective, since the seminal work by Krylov^28^ in the beginning of this century, the spin-flip (SF) method has emerged as a very attractive alternative to the MC approach in the electronic structure study of molecular species with a multireference character wave function. The SF methodology is based on the construction of electronic wave functions through promotions of α electrons into empty β spin-orbitals in combination with the use of a high-spin single determinant as the reference configuration. The SF idea has been implemented and applied with a variety of electronic structure formalisms: equation of motion coupled cluster (EOM-SF-CC),^29^ configuration interaction (SF-CI),^30^ and density functional theory (SF-DFT).^31^ In spite of the great success of these methods in the description of single bond dissociation processes,^32^ computation of singlet–triplet energy gaps in diradicals^33,34^ and doublet-quartet gaps in triradicals,^35^ the (standard) SF models also present some major drawbacks. The lack of spin completeness can result, in some unfavorable cases, in considerable spin contaminated wave functions.^36,37^ Also, their limited flexibilities have kept these models from tackling molecules with more than three strongly correlated electrons. While the spin incompleteness issue was fixed for the simplest SF model, that is SF-CI with singles (SF-CIS),^36,38^ there have been some early attempts in order to increase the number of simultaneous SF excitations^39^ as a strategy to capture larger amounts of static correlation. Finally, these ideas evolved into a new family of SF methods based on the definition of a restricted active space (RAS-SF).^40^ This method is spin complete by definition, and allows for the action of a SF operator with any number of concomitant α → β excitations. The RAS-SF approach has shown to be a computationally affordable and balanced approach to the study of ground and low-lying states with different spin multiplicities in molecules with some radical or multiradical character.^41–46^ Later, this methodology was generalized to the case of non-SF excitation operators: excitation energy (RAS-EE), electron affinity (RAS-EA), and ionization potential (RAS-IP) operators.^47^ Application of the RAS-XX family of methods is typically done within the hole and particle truncation of the excitation operator, which results in a rather flexible wave function with a low to moderate computational cost, which grows linearly both with the molecular and basis size. The main deficiency of the hole and particle approximation is the lack of effective dynamic correlation in the case of small to moderate active spaces. As a consequence, RASCI calculations, as other approaches suffering from the lack of dynamic correlation, such as CASSCF, might result in errors as large as 1–2 eV in the ground state relative energies of different atomic arrangements or between electronic states with rather different electronic structure nature. In 2014, Casanova^48^ introduced a second-order energy correction to the RAS-XX energy obtained with the hole and particle approximation, but the performance of this approach in the evaluation of excitation energies has never been seriously evaluated.

In the this study, we explore and discuss in detail the qualities and limitations of RASCI(2) schemes in the calculation of singlet and triplet excitation in organic molecules. The work is organized as follows. Section 2 briefly recalls the basic features of the RASCI methodology, the hole and particle approximation, and introduces the theoretical details of its second-order energy correction. Section 3 describes the computational details of the study. The obtained results and their discussion are presented in Section 4. Finally, the main achievements and results of the present work are summarized in Section 5.

## 2 Theoretical Background

### 2.1 The Hole and Particle Truncation of the Wave Function

In the RASCI scheme, the active orbital space, typically derived from a restricted Hartree–Fock (HF) solution, is partitioned into three subspaces: RAS1, RAS2, and RAS3, where RAS1 and RAS3 correspond to fully occupied and virtual orbitals, respectively, and RAS2 contain occupied and virtual frontier orbitals. The RASCI wave function is then constructed by applying an excitation operator (*R̂*) to the single HF reference configuration (ϕ_0_),

1 $$\left|\Psi \right\rangle =\mathrm{R̂}\left|\phi_{0}\right\rangle$$

where |Ψ⟩ is the target many-electron state.

Within the RASCI framework, the excitation operator is expanded in terms of excitations generating increasing number of holes and particles in RAS1 and RAS3, respectively (eq 2).

2 $$\mathrm{R̂}={\mathrm{r̂}}^{0}+{\mathrm{r̂}}^{1h}+{\mathrm{r̂}}^{1p}+{\mathrm{r̂}}^{1h1p}+{\mathrm{r̂}}^{2h}+{\mathrm{r̂}}^{2p}+{\mathrm{r̂}}^{2h1p}+{\mathrm{r̂}}^{1h2p}+{\mathrm{r̂}}^{2h2p}+\cdot \cdot \cdot$$

The term *r̂* ^0^ generates all possible configurations with a fixed number of electrons (*n*) distributed among the *m* orbitals of the RAS2 subspace, effectively a reduced full CI (FCI) within RAS2. The remaining terms in *R̂* describe excitations that involve *i*-holes in RAS1 and *j*-electrons (particles) in RAS3, indicated with superscripts *ihjp*.

To maintain a manageable computational cost, practical applications of RASCI often involve truncating the excitation operator *R̂* to include only the first three terms on the right-hand side of eq 2. This includes the FCI within RAS2 (*r̂* ^0^), along with all configurations involving one hole in RAS1 (*r̂* ^1*h*^), plus all terms with one electron in RAS3 (*r̂* ^1*p*^). The properties and limitations of this truncated approach, referred to as RASCI­(*h*,*p*) or simply as RAS­(*h*,*p*), have been thoroughly investigated in previous studies.^40,41,43,47,49^

### 2.2 Second-Order Perturbative Correction

One of the most remarkable limitations of the RAS­(*h*,*p*) approach is the lack of effective dynamic correlation, which notably impacts the accuracy of the ground state relative energies between different points of the potential energy surface, deteriorating for instance the computed binding energies and transition state barriers, and the accuracy of excitation energies, limiting its application in the study of photophysical and photochemical phenomena.^49^

The errors in state energies can be linked to the omitted higher-order terms, i.e., the hole and particle truncation in the excitation operator (eq 2). The impact of these contributions can be recovered through MRPT. Concretely, the second-order MRPT RASCI(2) method^48^ was introduced and implemented to correct RAS­(*h*,*p*) energies. In this method, a second-order energy functional for state *m* is defined as

3 $$E_{m}^{\mathrm{RASCI}\left(2\right)}=E_{m}^{\mathrm{RAS}\left(h,p\right)}-\sum_{k\neq H,P}\frac{|\left\langle k\right|Ĥ|0^{\left(m\right)}\rangle {|}^{2}}{E_{k}-E_{0}^{\left(m\right)}}$$

where the *m* index indicates the target state, *E*_*m*_^RAS(*h*,*p*)^ is the RAS­(*h*,*p*) energy, 0, *H*, and *P* refer to zeroth-order, one-hole, and one-particle configurations, respectively, *k* indices enumerate configurations beyond |0^(*m*)^⟩, and *E*_0_^(*m*)^ and *E*_*k*_ denote the energies of |0^(*m*)^⟩ and |*k*⟩, respectively.

#### 2.2.1 Zero-Order Excited-State Energies

At this stage, the unperturbed excited-state energies, denoted as {*E*_*k*_}, remain to be defined. In MRPT there is no unique or natural choice for {*E*_*k*_}, which has led to the development of various perturbation models. Within the Rayleigh–Schrödinger framework, two partitioning schemes are most commonly employed.

The first approach defines the excited-state energies as differences between orbital energies,

4 $$E_{k}=E_{0}^{\left(m\right)}+\Delta \varepsilon_{k}$$

where Δε_*k*_ are differences between occupied and virtual one-electron orbital energies. In a Møller–Plesset (MP)-like formulation, these quasiparticle energies are obtained as the eigenvalues of a generalized Fockian, which can be defined in two alternative forms,^50^

5 $$f_{\mathrm{pq}}^{1}=\sum_{r}h_{\mathrm{pr}}P_{\mathrm{rq}}+\sum_{\mathrm{rtv}}\left(\mathrm{pt}|\mathrm{rv}\right)\Gamma_{\mathrm{tv},\mathrm{qr}}$$

6 $$f_{\mathrm{pq}}^{2}=h_{\mathrm{pq}}+\sum_{\mathrm{rt}}P_{\mathrm{rt}}\left\langle \mathrm{pr}∥\mathrm{qt}\right\rangle$$

where *h*_*pq*_ is the one-electron Hamiltonian matrix element, and ⟨*pq*∥*rt*⟩ = (*pr*|*qt*) – (*pt*|*qr*) is the antisymmetric two electron integrals, using the (11|22) convention. The matrices *P* and Γ correspond to the one- and two-particle correlated density matrices, respectively. As a computationally simpler alternative to the full MP-type treatment, the diagonal elements of either *f* ^1^ or *f* ^2^ can be used directly as the one-electron orbital energies. This approximation is commonly referred to as the generalized Davidson–Kapuy (DK) partitioning scheme.^51^

The second widely used option is the Epstein–Nesbet (EN) partitioning,^52,53^ in which the excited-state energies are defined through the expectation value of the Hamiltonian with respect to the excited configuration.

7 $$E_{k}=\left\langle k\right|Ĥ\left|k\right\rangle$$

#### 2.2.2 Energy Level Shift

The introduction of an energy level shift (ε) is a common strategy in the implementation of second-order corrections to the energy of multiconfigurational wave functions, as shown in eq 8.

8 $$E_{m}^{\left(2\right)}=-\sum_{k}\frac{|\left\langle k\right|Ĥ|0^{\left(m\right)}\rangle {|}^{2}}{E_{k}-E_{0}^{\left(m\right)}+\varepsilon }$$

Incorporating ε into the second-order energy denominators often improves numerical stability and accuracy.^48^ This level shift helps mitigate divergences caused by weakly coupled intruder states and enhances convergence in iterative procedures. In fact, default values for ε, typically on the order of a few tenths of a Hartree, are implemented in several quantum chemistry packages.^54,55^ Moreover, denominator-dependent energy-level shifts have been introduced as effective strategies to address the intruder-state problem.^56,57^

In this work, we examine the use of one-electron energies obtained from the symmetric generalized Fockian *f* ^2^ (eq 6), employing the DK partitioning scheme. These results are compared to those obtained using the EN partitioning, with particular attention paid to the impact of the level-shift parameter. Optimization-based partitioning approaches within the Brillouin–Wigner framework,^58–60^ although relevant, fall outside the scope of this study and are not considered here.

## 3 Computational Details

The ground-state geometries of the 28 organic molecules investigated in this work were taken from Thiel and co-workers,^61^ who optimized them at the Møller–Plesset second-order perturbation theory (MP2) level with the double-ζ 6-31G­(d) basis set. Symmetry labels and molecular orientations follow Mulliken’s convention.^62^

RAS­(*h*,*p*) and RASCI(2) calculations of both singlet and triplet states employed the ground-state singlet HF wave function as the reference configuration, with the RAS2 space including the main orbitals required to describe both the ground and the target valence excited states. We note that the use of a low-spin HF reference in RAS­(*h*,*p*) may lead to a loss of size consistency in excitation energies for noninteracting fragments, as previously discussed.^49^ To assess the magnitude of this effect, we performed a size-intensivity test^63^ on a composite system consisting of two noninteracting fragments, ethylene and formaldehyde. The numerical results (Table S1) reveal only a modest deviation from strict size intensivity in the RAS­(*h*,*p*) and RASCI(2) excitation energies. Details of the chosen RAS2 spaces for each molecule are provided in the Supporting Information (Table S8). All calculations were carried out within the frozen-core approximation, with RAS1 containing the remaining doubly occupied orbitals and RAS3 comprising all virtual orbitals above RAS2. RAS­(*h*,*p*) and RASCI(2) results were compared against partially contracted N-electron valence state perturbation theory (PC-NEVPT2)^64^ calculations, using CASSCF reference wave functions with active spaces equivalent to the RAS2 spaces of the RAS­(*h*,*p*) computations. For brevity, we refer to these results as NEVPT2. Unless otherwise specified, excitation energies for all electronic structure methods were computed with the def2-TZVP basis set. Basis-set dependence was assessed through additional calculations with the 6-31G­(d) basis, and are provided in the Supporting Information (Tables S2 and S3).

All RAS­(*h*,*p*) and RASCI(2) calculations were performed with Q-Chem version 6.0.1,^65^ while NEVPT2 results were obtained from literature.^64^

## 4 Results and Discussion

In the following, we report the excitation energies computed using RASCI­(*h*,*p*) and second-order corrected RASCI(2) and NEVPT2 approaches for 72 singlet and 41 triplet valence excited states across a benchmark set of 28 organic molecules. The data set includes unsaturated aliphatic and aromatic hydrocarbons, heterocycles, conjugated aldehydes, ketones, amides, and nucleobases. From this point forward, results obtained using the two second-order perturbative partitioning schemes, Epstein–Nesbet and Davidson–Kapuy, are denoted as RAS­(EN) and RAS­(DK), respectively. Unless otherwise stated, RAS­(DK-ε) results refer to energies computed with a level shift of ε = 0.55 a.u. A detailed analysis of the dependence of RAS­(DK) excitation energies on the level-shift parameter (ε) is presented in Section 4.3. The computed excitation energies are compared to the theoretical best estimate values discussed in ref 61.

### 4.1 Vertical Transition Energies

The lowest excited singlet state of ethene, 1^1^ *B*_1*u*_, primarily arises from a single-electron excitation between the two frontier π-orbitals. The RAS­(*h*,*p*) method significantly overestimates this transition energy, by 1.6 eV compared to the best theoretical estimate (Table 1), highlighting the limitations of the hole and particle truncation in capturing essential electron correlation. Notably, this overestimation exceeds that observed at the CIS level (7.97 eV), despite RAS­(*h*,*p*) being a correlated method. This counterintuitive behavior can be rationalized by examining the dominant correlation effects in both the ground and excited states. The RAS­(*h*,*p*) model employs an active space with two electrons in the π (HOMO) and π* (LUMO) orbitals, which allows for inclusion of the LUMO double excitation (π*)^2^. This configuration substantially stabilizes the ground state relative to the Hartree–Fock reference. However, the excited state 1^1^ *B*_1*u*_ lacks access to higher-order correlation contributions in RAS­(*h*,*p*), leading to an overestimated excitation energy. In contrast, CIS neglects correlation effects in both the ground and excited states, resulting in a cancellation of errors and, consequently, a transition energy that fortuitously lies closer to the reference value. Second-order perturbative corrections using the DK and EN partitions significantly reduce the computed excitation gap. The RAS­(DK) result closely matches the best estimate, while RAS­(EN) overestimates the differential correlation energy. On the other hand, the level-shifted DK correction and NEVPT2 results both overestimate the excitation energy. For the lowest triplet state, 1^3^ *B*_1*u*_, which also corresponds to a π → π* excitation, correlation effects are considerably less pronounced than in the singlet manifold. As a result, the RAS­(*h*,*p*) method provides a reasonably accurate excitation energy (Table 2). In contrast, CIS underestimates the vertical triplet excitation (3.58 eV), primarily due to its neglect of ground-state correlation, which plays a more significant role in this case. The inclusion of second-order corrections with the DK partitioning leads to excellent agreement with the reference value, independent of the use of a level shift, further improving upon the RAS­(*h*,*p*) result. In analogy with the singlet case, RAS­(EN) overestimates the differential correlation energy, resulting in a triplet excitation energy that is approximately 0.4 eV too high.

**Table 1:** Vertical Transition Energies (in eV) to Excited Singlet States in Computed at the RASCI­(*h*,*p*), RASCI(2) with EN and DK Partitions, and NEVPT2 Levels with the def2-TZVP Basis Set^a^

molecule	state	RAS(*h*,*p*)	RAS(EN)	RAS(DK)	RAS(DK-ε)	NEVPT2	Best
ethene	1^1^ *B*_1*u*_ (π → π*)	9.40	6.93	7.88	8.41	8.64	7.80
butadiene	1^1^ *B*_*u*_ (π → π*)	7.61	5.06	6.06	6.57	6.21	6.18
	2^1^ *A*_*g*_ (π → π*)	6.66	7.44	6.43	6.63	6.80	6.55
hexatriene	2^1^ *A*_*g*_ (π → π*)	5.56	6.63	5.15	5.38	5.56	5.09
	1^1^ *B*_*u*_ (π → π*)	6.49	5.40	5.37	5.84	4.84	5.10
octatetraene	2^1^ *A*_*g*_ (π → π*)	4.85	6.14	4.25	4.52	4.72	4.47
	1^1^ *B*_*u*_ (π → π*)	5.81	4.49	4.58	5.01	4.04	4.66
cyclopropene	1^1^ *B*_1_ (σ → π*)	7.23	6.29	6.43	6.77	6.85	6.76
	1^1^ *B*_2_ (π → π*)	8.00	5.65	6.60	7.13	7.07	7.06
cyclopentadiene	1^1^ *B*_2_ (π → π*)	6.76	4.11	5.23	5.72	5.21	5.55
	2^1^ *A*_1_ (π → π*)	6.60	7.04	6.06	6.35	6.72	6.31
norbornadiene	1^1^ *A*_2_ (π → π*)	6.61	4.17	5.16	5.67	5.04	5.34
	1^1^ *B*_2_ (π → π*)	7.71	4.89	5.87	6.54	5.79	6.11
benzene	1^1^ *B*_2*u*_ (π → π*)	4.97	5.82	4.80	4.94	5.21	5.08
	1^1^ *B*_1*u*_ (π → π*)	7.35	5.81	6.18	6.56	6.40	6.54
naphthalene	1^1^ *B*_3*u*_ (π → π*)	4.42	4.55	3.69	3.99	4.37	4.24
	1^1^ *B*_2*u*_ (π → π*)	5.70	4.17	4.48	4.89	4.37	4.77
furan	1^1^ *B*_2_ (π → π*)	7.40	5.42	6.10	6.55	6.42	6.32
	2^1^ *A*_1_ (π → π*)	6.64	7.26	6.25	6.48	6.75	6.57
pyrrole	2^1^ *A*_1_ (π → π*)	6.41	6.99	6.08	6.29	6.56	6.37
	1^1^ *B*_2_ (π → π*)	7.29	6.00	6.24	6.64	6.78	6.57
imidazole	2^1^ *A*′ (π → π*)	6.80	7.02	6.23	6.50	6.80	6.19
	1^1^ *A*″ (*n* → π*)	7.14	7.03	6.26	6.59	6.97	6.81
pyridine	1^1^ *B*_1_ (*n* → π*)	5.60	4.97	4.54	4.91	5.26	4.59
	1^1^ *B*_2_ (π → π*)	5.18	5.74	4.84	5.03	5.33	4.85
	1^1^ *A*_2_ (*n* → π*)	6.32	5.38	4.97	5.44	5.46	5.11
	2^1^ *A*_1_ (π → π*)	7.43	6.21	6.34	6.70	7.09	6.26
pyrazine	1^1^ *B*_3*u*_ (*n* → π*)	4.65	4.19	3.80	4.08	4.20	3.95
	1^1^ *B*_2*u*_ (π → π*)	5.12	5.44	4.69	4.90	5.31	4.64
	1^1^ *A*_*u*_ (*n* → π*)	5.73	5.23	4.64	5.03	4.93	4.81
	1^1^ *B*_2*g*_ (*n* → π*)	5.98	5.90	5.14	5.45	5.86	5.56
	1^1^ *B*_1*u*_ (π → π*)	7.72	6.08	6.51	6.88	6.76	6.58
	1^1^ *B*_1*g*_ (*n* → π*)	7.29	7.19	6.09	6.54	6.77	6.60
pyrimidine	1^1^ *B*_1_ (*n* → π*)	5.20	4.44	4.09	4.46	4.52	4.55
	1^1^ *A*_2_ (*n* → π*)	5.71	4.83	4.50	4.91	4.81	4.91
pyridazine	1^1^ *B*_1_ (*n* → π*)	4.53	3.92	3.49	3.82	3.92	3.78
	2^1^ *A*_1_ (π → π*)	5.26	6.00	4.98	5.15	5.46	5.18
*s*-triazine	1^1^ *A*_1_^″^(*n* → π*)	5.49	4.56	4.21	4.63	4.65	4.60
	1^1^ *A*_2_^″^(*n* → π*)	5.31	4.94	4.27	4.63	4.88	4.66
	1^1^ *E*″ (*n* → π*)	5.45	4.78	4.28	4.67	4.87	4.71
	1^1^ *A*_2_^′^ (π → π*)	5.88	6.38	5.42	5.62	5.92	5.79
*s*-tetrazine	1^1^ *B*_3*u*_ (*n* → π*)	3.38	1.74	1.93	2.37	2.41	2.24
	1^1^ *B*_2*u*_ (π → π*)	5.18	6.07	4.88	5.05	5.47	4.91
formaldehyde	1^1^ *A*_2_ (*n* → π*)	4.49	3.85	3.79	4.03	4.22	3.88
	2^1^ *A*_1_ (π → π*)	10.13	8.84	9.12	9.42	8.79	9.30
acetone	1^1^ *A*_2_ (*n* → π*)	4.97	4.44	3.83	4.22	4.47	4.40
	2^1^ *A*_1_ (π → π*)	10.06	8.01	8.28	8.85	9.28	9.40
*p*-benzoquinone	1^1^ *B*_1*g*_ (*n* → π*)	3.46	3.24	2.04	2.53	3.00	2.78
	1^1^ *A*_*u*_ (*n* → π*)	3.58	3.38	2.09	2.62	2.99	2.80
	1^1^ *B*_3*g*_ (π → π*)	5.10	3.96	3.88	4.29	4.35	4.25
	1^1^ *B*_1*u*_ (π → π*)	6.36	4.11	4.84	5.33	4.85	5.29
formamide	1^1^ *A*″ (*n* → π*)	6.01	5.64	5.36	5.62	5.93	5.63
	2^1^ *A*′ (π → π*)	8.21	6.85	6.89	7.38	7.58	7.44
acetamide	1^1^ *A*″ (*n* → π*)	6.10	6.08	5.09	5.46	5.97	5.80
	2^1^ *A*′ (π → π*)	8.42	6.78	6.52	7.20	7.48	7.27
propanamide	1^1^ *A*″ (*n* → π*)	6.20	6.08	4.96	5.42	5.99	5.72
cytosine	2^1^ *A*′ (π → π*)	5.35	4.36	3.95	4.45	4.70	4.66
	1^1^ *A*″ (*n* → π*)	5.65	5.87	4.27	4.77	5.50	4.87
	2^1^ *A*″ (*n* → π*)	6.14	6.08	4.42	5.05	5.73	5.26
	3^1^ *A*′ (π → π*)	6.53	5.76	4.89	5.49	5.65	5.62
thymine	1^1^ *A*″ (*n* → π*)	5.58	5.40	3.89	4.50	4.96	4.82
	2^1^ *A*′ (π → π*)	6.28	4.16	4.51	5.12	5.05	5.20
	2^1^ *A*″ (*n* → π*)	7.03	6.62	5.31	5.92	6.49	6.16
	3^1^ *A*′ (π → π*)	7.50	5.86	5.33	6.12	6.32	6.27
uracil	1^1^ *A*″ (*n* → π*)	5.37	5.65	3.91	4.45	4.92	4.80
	2^1^ *A*′ (π → π*)	6.28	4.64	4.69	5.24	5.27	5.35
	2^1^ *A*″ (*n* → π*)	6.94	6.55	5.35	5.92	6.42	6.10
	3^1^ *A*′ (π → π*)	7.32	6.36	5.47	6.15	6.22	6.26
adenine	1^1^ *A*″ (*n* → π*)	6.34	5.27	4.43	5.09	5.36	5.12
	2^1^ *A*′ (π → π*)	5.98	4.95	4.46	5.01	5.43	5.25
	3^1^ *A*′ (π → π*)	6.28	4.69	4.82	5.33	5.07	5.25
	2^1^ *A*″ (*n* → π*)	7.05	5.64	5.10	5.77	6.07	5.75

a RAS­(DK-ε) refers to excitation energies obtained with a ε = 0.55 a.u. level shift.

**Table 2:** Vertical Transition Energies (in eV) to Excited Triplet States in Computed at the RASCI­(*h*,*p*), RASCI(2) with DK and EN Partitions, and NEVPT2 Levels with the def2-TZVP Basis Set^a^

molecule	state	RAS(*h*,*p*)	RAS(EN)	RAS(DK)	RAS(DK-ε)	NEVPT2	Best
ethene	1^3^ *B*_1*u*_ (π → π*)	4.26	4.91	4.46	4.49	4.60	4.50
butadiene	1^3^ *B*_*u*_ (π → π*)	3.27	3.82	3.18	3.27	3.38	3.20
	1^3^ *A*_*g*_ (π → π*)	4.99	5.85	5.03	5.11	5.27	5.08
hexatriene	1^3^ *B*_*u*_ (π → π*)	2.71	3.36	2.50	2.62	2.73	2.40
	1^3^ *A*_*g*_ (π → π*)	4.24	5.30	4.14	4.25	4.39	4.15
octatetraene	1^3^ *B*_*u*_ (π → π*)	2.35	3.10	2.04	2.18	2.32	2.20
	1^3^ *A*_*g*_ (π → π*)	3.68	4.88	3.44	3.57	3.72	3.55
cyclopropene	1^3^ *B*_2_ (π → π*)	4.25	4.70	4.23	4.33	4.54	4.34
	1^3^ *B*_1_ (σ → π*)	6.79	6.18	6.15	6.45	6.58	6.62
cyclopentadiene	1^3^ *B*_1_ (π → π*)	3.20	3.77	3.11	3.21	3.32	3.25
	1^3^ *A*_1_ (π → π*)	4.94	5.80	4.85	4.97	5.22	5.09
norbornadiene	1^3^ *A*_2_ (π → π*)	3.76	4.14	3.31	3.56	3.79	3.72
	1^3^ *B*_2_ (π → π*)	4.13	4.87	3.70	3.96	4.30	4.16
benzene	1^3^ *B*_1*u*_ (π → π*)	3.89	5.04	3.99	4.04	4.32	4.15
	1^3^ *E*_1*u*_ (π → π*)	4.81	5.46	4.61	4.74	4.98	4.86
naphthalene	1^3^ *B*_2*u*_ (π → π*)	3.09	3.77	2.76	2.92	3.26	3.11
	1^3^ *B*_3*u*_ (π → π*)	4.26	4.52	3.64	3.89	4.24	4.18
furan	1^3^ *B*_2_ (π → π*)	3.96	4.79	4.02	4.07	4.33	4.17
	1^3^ *A*_1_ (π → π*)	5.28	6.27	5.28	5.37	5.62	5.48
pyrrole	1^3^ *B*_2_ (π → π*)	4.31	5.13	4.37	4.42	4.73	4.48
	1^3^ *A*_1_ (π → π*)	5.37	6.18	5.29	5.40	5.68	5.51
imidazole	1^3^ *A*′ (π → π*)	4.64	5.37	4.58	4.66	4.77	4.69
	2^3^ *A*′ (π → π*)	5.75	6.49	5.59	5.70	5.89	5.79
	1^3^ *A*″ (*n* → π*)	6.65	6.69	5.87	6.17	6.46	6.37
pyridine	1^3^ *A*_1_ (π → π*)	4.12	5.15	4.14	4.21	4.47	4.06
	1^3^ *B*_1_ (*n* → π*)	5.08	4.54	4.05	4.41	4.58	4.25
	1^3^ *B*_2_ (π → π*)	4.87	5.20	4.53	4.69	4.58	4.64
	2^3^ *A*_1_ (π → π*)	5.02	5.60	4.77	4.92	5.13	4.91
*s*-tetrazine	1^3^ *B*_3*u*_ (*n* → π*)	2.76	1.29	1.33	1.77	1.64	1.89
formaldehyde	1^3^ *A*_2_ (π → π*)	4.00	3.50	3.39	3.47	3.75	3.50
	1^3^ *A*_1_ (π → π*)	5.73	6.43	5.94	5.87	6.06	5.87
acetone	1^3^ *A*_2_ (*n* → π*)	4.55	4.24	3.54	3.89	4.10	4.05
	1^3^ *A*_1_ (π → π*)	6.11	6.66	5.56	5.81	6.06	6.03
*p*-benzoquinone	1^3^ *B*_1*g*_ (*n* → π*)	3.21	3.02	1.86	2.32	2.82	2.51
	1^3^ *A*_*u*_ (*n* → π*)	3.35	3.15	1.90	2.41	2.82	2.62
formamide	1^3^ *A*″ (*n* → π*)	5.68	5.38	5.08	5.32	5.64	5.36
	1^3^ *A*′ (π → π*)	5.85	5.92	5.51	5.69	5.81	5.74
acetamide	1^3^ *A*″ (*n* → π*)	5.78	5.88	4.85	5.20	5.52	5.42
	1^3^ *A*′ (*n* → π*)	6.23	6.48	5.40	5.73	5.63	5.88
propanamide	1^3^ *A*″ (*n* → π*)	5.84	5.91	4.71	5.14	5.54	5.45
	1^3^ *A*′ (π → π*)	6.49	6.49	5.26	5.74	5.86	5.90

a RAS­(DK-ε) refers to excitation energies obtained with a ε = 0.55 a.u. level shift.

In the three polyenes studied, butadiene, hexatriene, and octatetraene, the π → π* transition to the 1^1^ *B*_*u*_ state is significantly overestimated by RAS­(*h*,*p*), with deviations of around 1.4 eV, similar to the overestimation observed for ethene. This discrepancy is substantially corrected by the RAS­(DK) method, specially with ε = 0, which yields excitation energies with errors comparable to those from NEVPT2. In contrast, RAS­(EN) results show much larger deviations. The lowest excited singlet state of the same symmetry as the ground state, 2^1^ *A*_*g*_, presents a particularly demanding case for electronic structure methods due to its strong multiconfigurational character, most notably the significant contribution from the two-electron HOMO-to-LUMO double excitation. RAS­(*h*,*p*) also overestimates the excitation energy to this state, but the errors are noticeably smaller than for the 1^1^ *B*_*u*_ transition. The DK correction further improves the accuracy, achieving deviations within 0.1–0.3 eV relative to the best estimates. On the other hand, RAS­(EN) performs poorly, especially for butadiene and hexatriene, significantly overestimating this excitation. It is worth noticing that excitation energies to 2^1^ *A*_*g*_ exhibit a weaker dependence on the level shift parameter than in 1^1^ *B*_*u*_. A key quantity in the photophysical characterization of these systems is the relative energy between the 1^1^ *B*_*u*_ and 2^1^ *A*_*g*_ states. RAS­(DK), and to a lesser extend RAS­(DK-ε), capture this energy gap with high accuracy, reproducing the correct energetic ordering and magnitude, underscoring its reliability in describing low-lying singlet excitations in polyenes. The two lowest triplet states in polyenes, 1^3^ *B*_*u*_ and 1^3^ *A*_*g*_, are well separated in energy, with 1^3^ *B*_*u*_ lying significantly lower. The RAS­(*h*,*p*) method yields highly accurate excitation energies for both states, and these results are maintained, or slightly refined, by the RAS­(DK) corrections. In contrast, RAS­(EN) markedly overestimates the energies of both triplet states, deteriorating the already satisfactory performance of RAS­(*h*,*p*) and confirming its tendency to overshoot differential correlation effects in triplet manifolds.

The lowest excited singlet in cyclopropene corresponds to a σ → π* valence transition, with the 1^1^ *B*_1_ (π → π*) state located 0.3 eV higher in energy. This state ordering is correctly reproduced by all methods reported in Table 1, except for RAS­(EN), which fails to capture the excitation energies accurately, most notably for the 1^1^ *B*_2_ state, whose vertical gap is underestimated by about 1.4 eV. While RAS­(*h*,*p*) significantly overestimates the excitation energies of both singlet states, the inclusion of second-order DK-based corrections leads to results that are in good agreement with the best available theoretical benchmarks, specially with ε = 0.5 a.u. The corresponding triplet states exhibit an inverted energy ordering compared to the singlets, with 1^3^ *B*_1_ lying below 1^3^ *B*_2_. All methods yield reasonably accurate excitation energies for these states, with RAS­(*h*,*p*) and RAS­(DK-ε) performing particularly well.

The two lowest singlet excitations in cyclopentadiene closely mirror those observed in polyenes: the 1^1^ *B*_1_ state, dominated by a single-electron π → π* transition, lies significantly below the 2^1^ *A*_1_ state, which exhibits strong multiconfigurational character due to important double excitation contributions. RAS­(*h*,*p*) substantially overestimates the excitation energy to 1^1^ *B*_1_, incorrectly predicting it to lie above 2^1^ *A*_1_, thus inverting the correct state ordering. In contrast, RAS­(DK) and RAS­(DK-ε) significantly improve the description, yielding excitation energies in good agreement with benchmark values, consistent with its performance in polyenes. Triplet excitation energies are accurately reproduced by RAS­(*h*,*p*) and the DK partitioning, although the DK correction without a level shift slightly worsens agreement with the reference values. In contrast, applying a level shift of ε = 0.55 a.u. yields highly accurate results.

For norbornadiene, the excitation energy to the lowest singlet–singlet transitions are significantly overestimated by RAS­(*h*,*p*), with errors exceeding 1 eV. Second-order perturbative corrections markedly decrease excitation energies, with RAS­(DK) yielding results in excellent agreement with reference values, slightly outperforming NEVPT2 in this case. In contrast, RAS­(EN) overcorrects, leading to an overestimation of dynamic correlation effects and thus excessive lowering of the excitation energy. The behavior for triplet excitations is more favorable at the RAS­(*h*,*p*) level, which provides remarkably accurate energies. The application of DK and EN corrections slightly degrades the agreement with reference data in this case.

RAS­(*h*,*p*) yields reasonably accurate excitation energies for the lowest singlet state in both benzene and naphthalene, but significantly overestimates the energy of the second excited singlet. This overestimation is substantially corrected by the RASCI(2) methods, which improve the description of the higher singlet while maintaining only moderate deviations for the lowest one. As observed in some of the previous cases, the most accurate triplet excitation energies are obtained at the RAS­(*h*,*p*) level.

The two lowest excited singlet states in furan and pyrrole closely resemble those of polyenes. The single-electron π → π* excitation (1^1^ *B*_2_) is significantly overestimated by RAS­(*h*,*p*), whereas the method yields accurate excitation energies for the first singlet state of totally symmetric character within the C_2*v*_ point group. RASCI(2) methods substantially reduce the excitation energy to the 1^1^ *B*_2_ state, with the DK partition showing particularly good agreement with reference data. In contrast, vertical excitation energies to the lowest triplet states are already well captured by the uncorrected RAS­(*h*,*p*) method and are slightly improved with the DK corrections, while the EN partition noticeably worsens the accuracy of the triplet energies.

The lowest excited singlet state in imidazole, of π → π* character, is overestimated by RAS­(*h*,*p*), RAS­(EN), and NEVPT2, whereas RAS­(DK), and to a lesser extend RAS­(DK-ε), yields a transition energy in much closer agreement with the best available computational estimate. The second singlet–singlet excitation, involving an *n* → π* transition, is slightly overestimated by RAS­(*h*,*p*), improved by RAS­(EN), RAS­(DK-ε) and NEVPT2, and somewhat overstabilized by RAS­(DK). Excitation energies to the lowest triplet states are generally well captured by all approaches, particularly for *A*′ (π → π*) states. The largest deviation arises in the RAS­(EN) prediction for the lowest triplet, which noticeably overestimates its energy.

The computed excitation energies to singlet states in six-membered N-heterocycles: pyridine, pyrazine, pyrimidine, pyridazine, *s*-triazine, and *s*-tetrazine, are systematically overestimated by RAS­(*h*,*p*), with particularly large errors observed for *n* → π* transitions, though π → π* excitations are also significantly affected. These overestimations are substantially corrected by RAS­(DK) and RAS­(DK-ε), which yield excitation energies in excellent agreement with reference values and average errors comparable to those from NEVPT2, or even smaller when ε = 0.55 a.u. However, while NEVPT2 and RAS­(DK-ε) generally exhibit a slight tendency to overestimate excitation energies, RAS­(DK) tends to slightly underestimate them. In terms of excitation type, RAS­(DK) performs marginally better for π → π* transitions, whereas RAS­(DK-ε) offers improved accuracy for *n* → π* excitations. The strong performance of RAS­(DK) and specially RAS­(DK-ε) is not matched by RAS­(EN), which, despite improving upon RAS­(*h*,*p*), exhibits consistently larger deviations from benchmark results. As in previous cases, RAS­(*h*,*p*) accurately captures the energy gaps to low-lying π → π* triplet states in pyridine, with only a slight overestimation. This level of accuracy is maintained (or even slightly improved) with RAS­(DK), which typically reverses the sign of the small RAS­(*h*,*p*) errors, leading to mild underestimations. Results with RAS­(DK-ε) recover the right triplet state order and are in excellent numerical agreement with best estimates. In contrast, RAS­(EN) consistently overestimates these triplet excitation energies. For the lowest *n* → π* triplet state in pyridine and *s*-tetrazine, the RAS­(*h*,*p*) values overestimate the excitation energies by more than 0.8 eV. These large errors are substantially mitigated by the second-order perturbative corrections, particularly with the DK scheme, though the results with ε = 0 a.u. tend to slightly underestimate the excitation energies.

Second-order perturbative corrections yield excitation energies to the low-lying states of formaldehyde in good agreement with the best available estimates, effectively correcting the systematic overestimation observed with RAS­(*h*,*p*) for all cases except the ^3^ *A*_1_ (π → π*) state. The DK approaches, in particular, provide highly accurate results, with typical deviations around 0.1 eV. However, the performance of RAS­(DK) is less satisfactory in the case of acetone. Although RAS­(*h*,*p*) again overestimates the excitation energies, RAS­(DK) systematically overcorrects these values, leading to significant underestimations, for example, an error of 1.12 eV for the 2^1^ *A*_1_ (π → π*) transition. The use of a level shift largely improves the DK results, with RAS­(DK-ε) producing rather accurate transition energies. A similar trend is observed for *p*-benzoquinone, where all low-lying singlet and triplet excitations are overestimated by RAS­(*h*,*p*) and underestimated by RAS­(DK), indicating a systematic overcorrection. In contrast, RAS­(DK-ε) provides excitation energies in excellent agreement with the best reference values. Results obtained with RAS­(EN), while exhibiting average deviations comparable to those of RAS­(DK) across the low-lying transitions of formaldehyde and the two ketones, display a less consistent error pattern, lacking the systematic trends observed in RAS­(DK).

Similar trends are observed for the RAS­(*h*,*p*) and second-order perturbative approaches in the low-lying excitations of the three studied amides (formamide, acetamide, and propanamide). RAS­(*h*,*p*) consistently overestimates excitation energies, while RAS­(DK) tends to underestimate them. RAS­(EN) yields improved average deviations; however, its performance is less consistent, with a tendency to slightly overestimate *n* → π* transitions, underestimate π → π* singlet excitations, and overestimate triplet energies. As in the case of aldehydes and ketones, RAS­(DK-ε) provides the most accurate results, even surpassing the performance of NEVPT2.

Finally, we assess the performance of RAS­(DK) and RAS­(EN) in computing excitation energies to low-lying singlet states in four nucleobases: cytosine, thymine, uracil, and adenine. As in previous cases, RAS­(*h*,*p*) systematically overestimates the transition energies. NEVPT2 and specially RAS­(DK-ε), by contrast, yield consistently accurate results across all systems. RAS­(DK) tends to underestimate excitation energies, with errors ranging from 0.4 to 0.9 eV, irrespective of the nature of the transition (π → π* or *n* → π*). While RAS­(EN) gives somewhat smaller average deviations than RAS­(DK), its performance is less systematic and lacks a consistent trend relative to the reference data.

### 4.2 Statistical Evaluation of Excitation Energies

A statistical assessment of the performance of the different approaches for computing singlet excitation energies is presented in Table 3. The results clearly illustrate both the strengths and limitations of the hole and particle truncation in the RASCI wave function for computing excitation energies. Statistical analysis reveals a systematic and substantial overestimation of singlet excitation energies, with a mean error of 0.8 eV and deviations reaching up to 1.6 eV.

**Table 3:** Statistical Analysis of the Errors for the Singlet Transition Energies Listed in Table 1 ^a^

	RAS(*h*,*p*)	RAS(EN)	RAS(DK)	RAS(DK-ε)	NEVPT2
MSE	0.77	0.14	– 0.40	0.02	0.15
MAE	0.77	0.66	0.42	0.18	0.26
RMSE	0.86	0.77	0.50	0.23	0.32
SD	0.38	0.76	0.30	0.23	0.29
Max(+)	1.60	1.67	0.27	0.74	0.84
Max( – )	– 0.11	– 1.44	– 1.12	– 0.55	– 0.62
R	0.96	0.80	0.97	0.98	0.97

a MSE: mean signed error; MAE: mean absolute error; RMSE: root-mean-square error; SD: standard deviation; Max­(+): largest positive deviation; Max(−): largest negative deviation; R: linear correlation coefficient.

Including higher-order hole and particle contributions up to 2*h*2*p* via the EN partitioning moderately reduces the mean error, while introducing greater dispersion in the results: the standard deviation doubles, and the linear correlation with reference values worsens considerably compared to RAS­(*h*,*p*).

On the other hand, although RAS­(DK) does not match the accuracy of the well-established NEVPT2 approach, it represents a clear improvement over RAS­(*h*,*p*), reducing the average error by roughly a factor of 2. Moreover, unlike RAS­(EN), the DK partition yields much better statistical indicators, including a low standard deviation and a correlation coefficient close to unity. The remaining deviations in RAS­(DK) singlet excitation energies are primarily due to systematic overcorrection relative to RAS­(*h*,*p*), leading to a consistent underestimation of the transition energies. This bias can be effectively mitigated by introducing a level shift. With ε = 0.55 a.u., the average error is reduced to below 0.2 eV, surpassing the accuracy of NEVPT2. Statistical analysis confirms the strong and robust performance of RAS­(DK-ε), showing both a small standard deviation and an excellent linear correlation with the reference values.

RAS­(*h*,*p*) triplet excitation energies are considerably more accurate than their singlet counterparts, showing smaller average errors that mainly arise from a slight overestimation of the transition energies (Table 4). The performance of RAS­(EN) for triplet states mirrors its behavior for singlets, representing in this case a deterioration relative to RAS­(*h*,*p*). In contrast, RAS­(DK) yields errors comparable to those of the hole and particle approximation, but with a slight tendency to underestimate triplet excitation energies. Applying a level shift, RAS­(DK-ε) achieves excellent agreement with the best estimates, delivering an overall performance very similar to that of NEVPT2.

**Table 4:** Statistical Analysis of the Errors for the Triplet Transition Energies Listed in Table 2 ^a^

	RAS(*h*,*p*)	RAS(EN)	RAS(DK)	RAS(DK-ε)	NEVPT2
MSE	0.15	0.54	–0.27	–0.08	0.13
MAE	0.24	0.59	0.28	0.12	0.16
RMSE	0.33	0.65	0.35	0.14	0.18
SD	0.30	0.36	0.23	0.12	0.13
Max(+)	0.87	1.33	0.10	0.22	0.41
Max(−)	–0.26	–0.60	–0.74	–0.31	–0.25
*R*	0.97	0.95	0.98	1.00	0.99

a MSE: mean signed error; MAE: mean absolute error; RMSE: root-mean-square error; SD: standard deviation; Max­(+): largest positive deviation; Max(−): largest negative deviation; *R*: linear correlation coefficient.

### 4.3 Impact of the Energy Level Shift

In the following, we examine the impact of the energy level-shift parameter on the computed excitation energies. Our analysis focuses on singlet and triplet transitions obtained with the DK partition, which, as discussed above, shows superior statistical performance compared to the EN partition. In addition to smaller average errors, DK results display reduced standard deviations and stronger linear correlations with the best estimates. The dependence of EN excitation energies on the level shift is provided in the Supporting Information (Figures S1 and S2).

Overall, introducing a level shift systematically improves the performance of RAS­(DK) by mitigating the overestimated contribution of the second-order correction through an increased denominator in eq 8. The dependence of the MAE on ε for all computed excitations (Figure 1) indicates an optimal range of 0.5–0.7 a.u. for low-lying states. When focusing solely on singlet excitations, the optimal value is approximately 0.55 a.u., the value used in Tables 1 and 2, whereas triplet states achieve their best accuracy at slightly larger ε values. This difference reflects the stronger overestimation of singlet excitation energies by RAS­(*h*,*p*) relative to their triplet counterparts. In practice, a level shift around 0.5 a.u. offers a good compromise, delivering accurate excitation energies and reliable singlet–triplet gaps.

**Figure 1:**
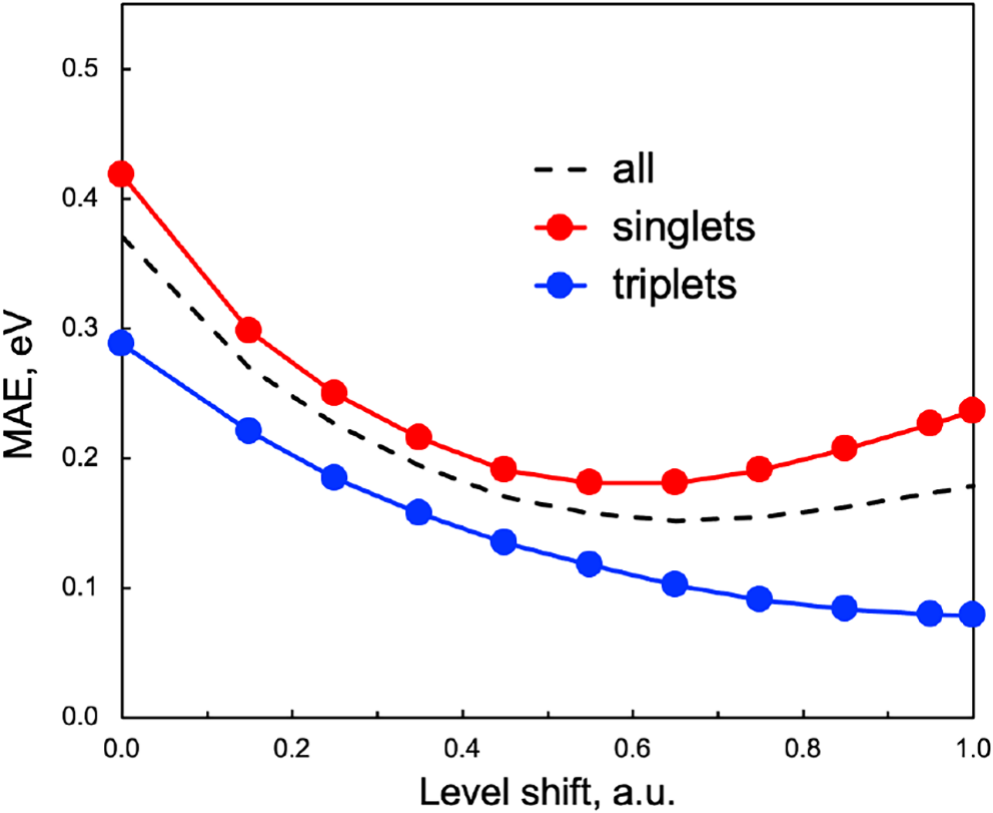
Dependence of the mean absolute error (MAE, in eV) on the level-shift parameter (ε, in a.u.) for RASCI(2) with the DK partition: all excitations (dashed black line), triplet states (blue), and singlet states (red).

In addition to spin symmetry, the sensitivity of excitation energies to electron correlation effects also depends on the orbital character of the transition. This is illustrated in Figure 2a, where the optimal ε value is smaller for ^1^ ππ* excitations (ε ≈ 0.45 a.u.) than for ^1^ nπ* excitations (ε ≈ 0.70 a.u.). This difference reflects the stronger influence of dynamic electron correlation on the computed energies of ^1^ ππ* states.

**Figure 2:**
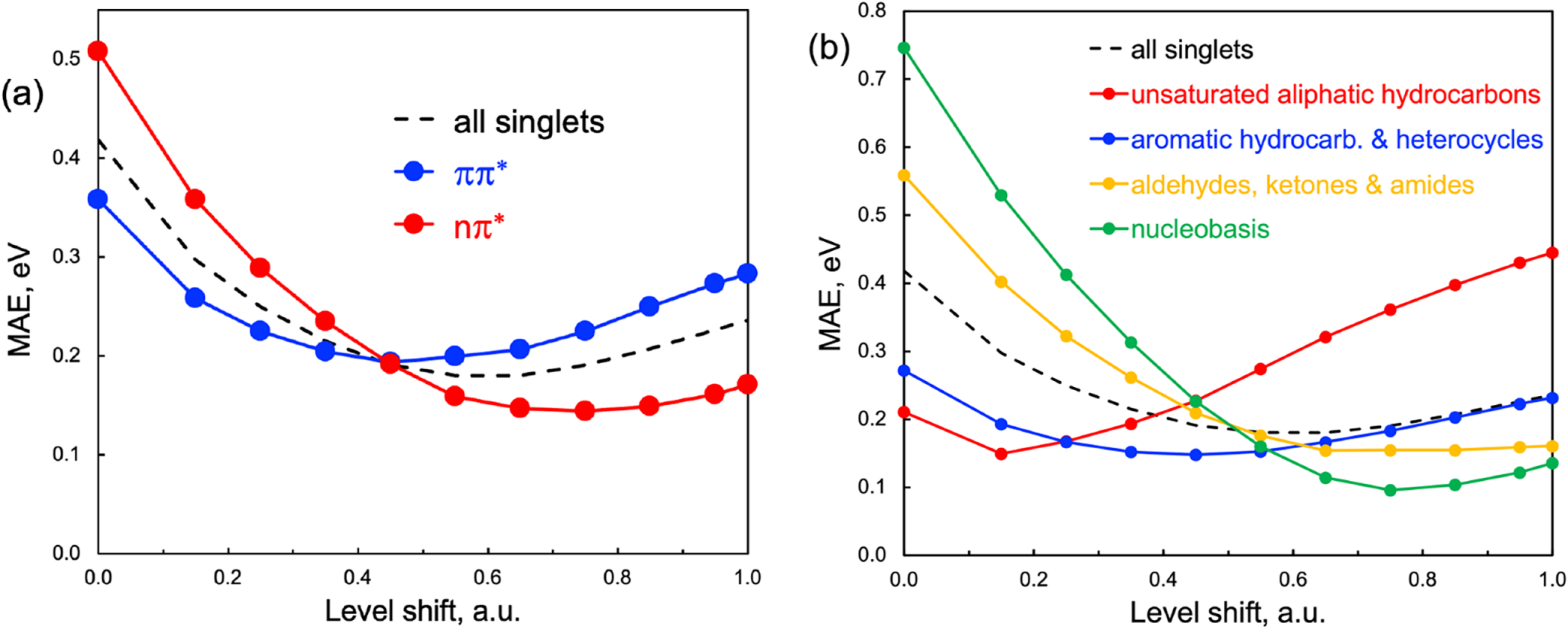
Dependence of the mean absolute error (MAE, in eV) on the level-shift parameter (ε, in a.u.) for RASCI(2) with the DK partition: (a) all singlets (dashed black line), ππ* singlet states (blue), and nπ* singlet states (red); and (b) singlet excitations grouped by different molecular families.

When the results are grouped by molecular family, distinct trends emerge in the dependence of excitation energy errors on the level-shift parameter (Figure 2b). Consistent with the observations of Schreiber et al. for CASPT2 excitation energies, singlet excited states in polyenes are best reproduced by RAS­(DK) with relatively small level shifts (ε < 0.2 a.u.). In contrast, for aldehydes, ketones, amides, and nucleobases, the optimal performance is achieved with larger shifts (ε ≥ 0.6 a.u.). Aromatic hydrocarbons and heterocycles show a much weaker sensitivity to ε, with an optimal value around 0.5 a.u.

### 4.4 Second-Order Energy Contributions

The second-order perturbation scheme in RASCI(2) enables a detailed breakdown of electron correlation contributions, categorized by the excitation rank in terms of hole and/or particle operators. This is achieved by reorganizing the second-order energy corrections in the right-hand side of eq 3 into distinct classes beyond those included in the RAS­(*h*,*p*) approximation, with the expansion to excitations involving up to two holes and/or two particles. These classes include 1*h*1*p*, 2*h*, 2*p*, 2*h*1*p*, 1*h*2*p*, and 2*h*2*p* terms. Such decomposition provides a detailed view of the excitation ranks that contribute most significantly to the correlation energy and their relative impact on excitation energies.

We begin by analyzing the ground-state correlation energy contributions. Figure 3 shows the average total ground-state correlation energy per electron pair, along with the individual contributions obtained using the RAS­(DK-ε) scheme. The average total correlation energy per electron pair captured by the second-order perturbative correction is ∼1 eV, in agreement with the typical dynamic correlation energies obtained in small and medium molecules. Among the different contributions, 2*h*2*p* is the one with the largest weight, followed by 1*h*2*p*, while 2*h* and 2*h*1*p* have minor contributions, especially the former. The dominant role of the 2*h*2*p* and 1*h*2*p* components can be attributed to their significantly larger number of possible contributions. This number scales quadratically with both the size of RAS1 (proportional to the number of electrons in the system) and the size of RAS3 (proportional to the number of basis functions).

**Figure 3:**
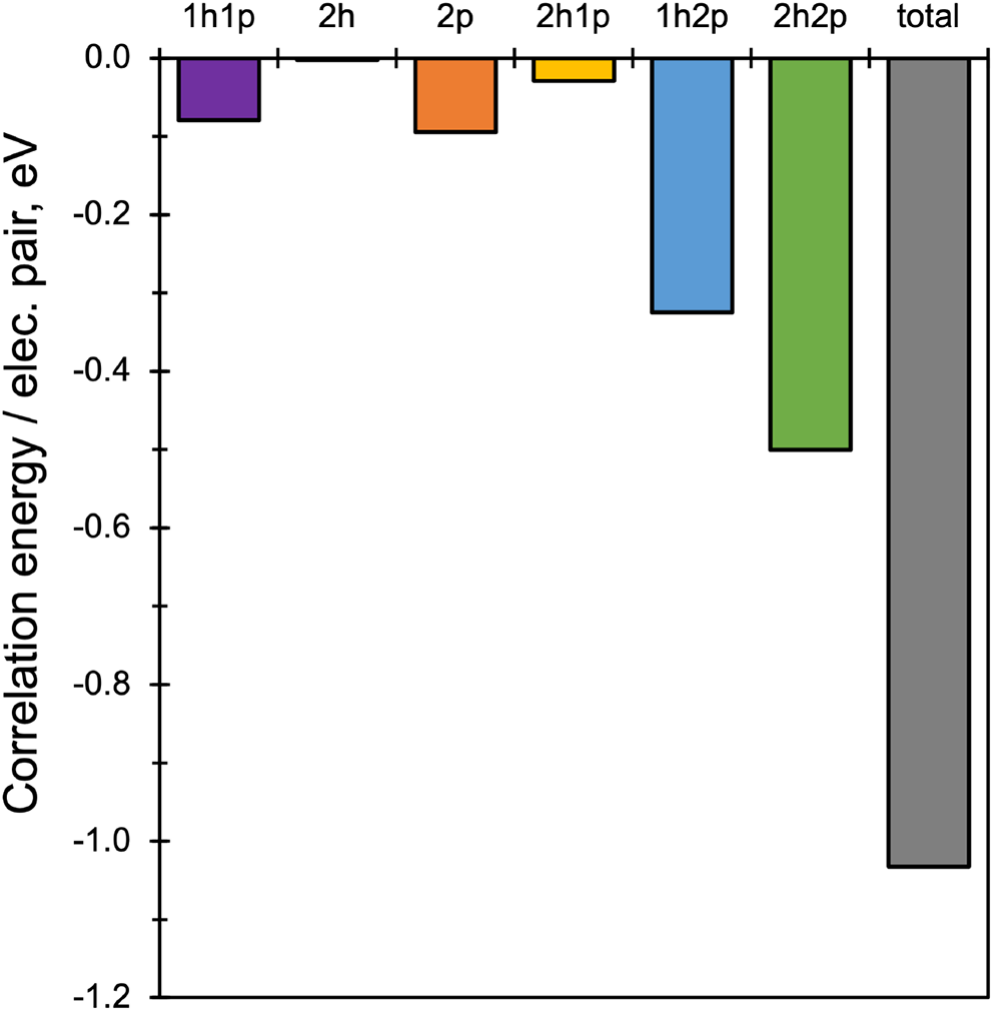
Average correlation energy contributions (in eV) per electron pair to the ground state of the studied molecules obtained at the RAS­(DK-ε)/def2-TZVP level.

The correction of RAS­(*h*,*p*) excitation energies by second-order perturbative schemes arises from the differential correlation energy, the difference between the correlation energies of the ground and excited states. Figure 4 displays the average differential correlation energy per electron pair for singlet and triplet excited states obtained with RAS­(DK-ε). On average, these corrections are negative, leading to a systematic reduction of excitation energies: approximately 1 eV for low-lying singlet states and about 0.3 eV for triplet states.

**Figure 4:**
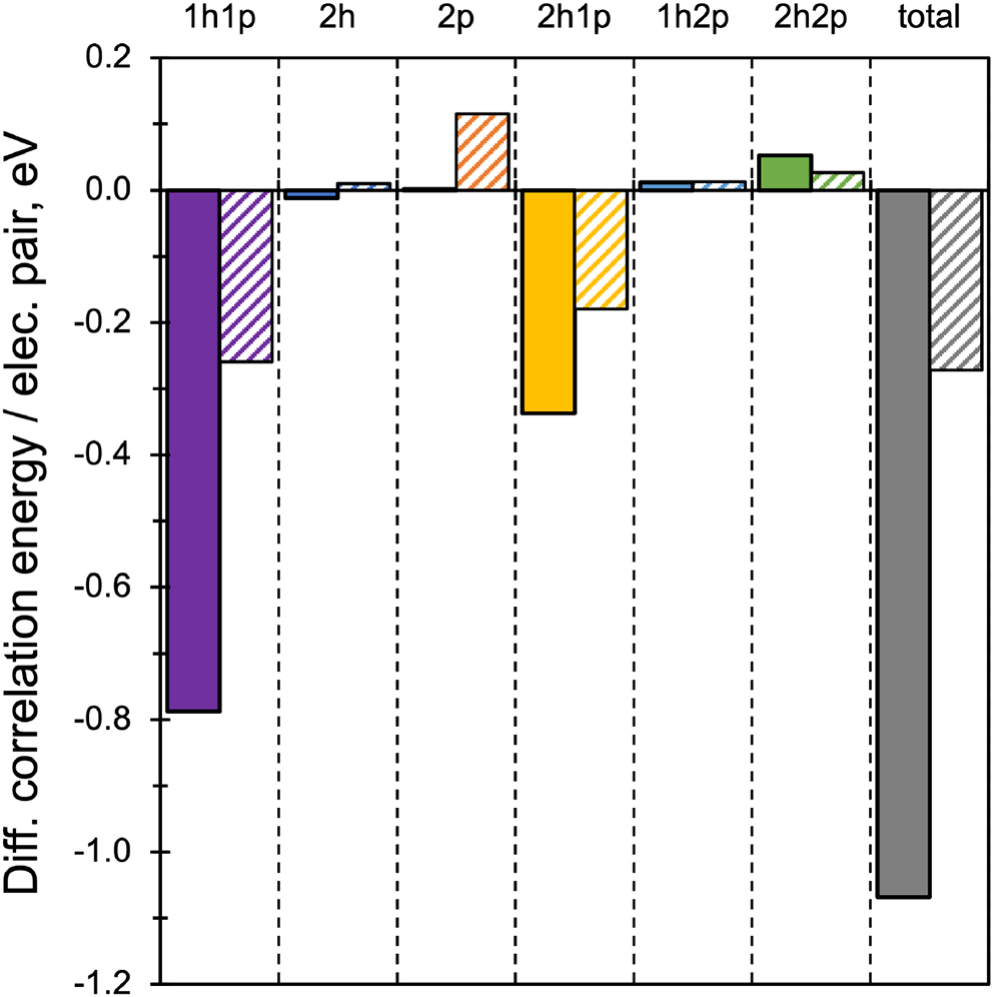
Average differential correlation energy contributions (in eV) per electron pair of excited singlet (solid bars) and triplet (diagonal striped bars) of the studied molecules obtained at the RAS­(DK-ε)/def2-TZVP level.

Although the 1*h*2*p* and 2*h*2*p* components involve the largest number of terms, their contributions to excitation energy corrections are minimal, indicating that their magnitudes are similar in both ground and excited states. The dominant effect comes from the 1*h*1*p* term, which lowers singlet and triplet excitation energies by roughly 0.8 and 0.3 eV, respectively. Like the variational 1*h* and 1*p* excitations in RAS­(*h*,*p*), these 1*h*1*p* terms likely correspond to state-specific orbital relaxation effects, as they represent single excitations relative to the dominant configurations in the zero-order wave function.^66^ The 2*h*1*p* terms also make a significant contribution, especially for singlet states. For triplets, their effect is partially offset by the positive contributions from 2*h*, 2*p*, 1*h*2*p*, and 2*h*2*p* components.

Interestingly, the most influential differential terms (1*h*1*p* and 2*h*1*p*) are much less computationally demanding than the 1*h*2*p* and 2*h*2*p* terms. This observation suggests that the latter could be omitted when computing excitation energies, potentially reducing cost with minimal accuracy loss. Indeed, excluding the most expensive 2*h*2*p* terms has a negligible impact on both singlet and triplet excitation energies (Tables S5–S7). Alternatively, one might explore scaling strategies to further improve accuracy,^67–72^ though this avenue lies beyond the scope of the present work.

### 4.5 Basis Set Comparison

In this section, we examine the basis set dependence of the computed excitation energies. Specifically, we compare the results obtained so far with the def2-TZVP basis set to those calculated using the smaller 6-31G­(d) basis. Table 5 summarizes the performance of RAS­(*h*,*p*) and RAS­(DK-ε) for valence singlet-state excitation energies with the two basis sets. The corresponding comparison for triplet states is provided in the Supporting Information (Table S4).

**Table 5:** Statistical Analysis of the Errors for the Singlet Transition Energies Listed in Table 1 Obtained with RAS­(*h*,*p*) and RAS­(DK-ε), and with 6-31G­(d) and def2-TZVP Basis Sets^a^

	RAS(*h*,*p*)		RAS(DK-ε)	
	6-31G(d)	def2-TZVP	6-31G(d)	def2-TZVP
MSE	0.90	0.77	0.15	0.02
MAE	0.91	0.77	0.27	0.18
RMSE	1.05	0.86	0.36	0.23
SD	0.54	0.38	0.33	0.23
Max(+)	2.67	1.60	1.37	0.74
Max(−)	–0.09	–0.11	–0.39	–0.55
R	0.94	0.96	0.97	0.98

a MSE: mean signed error; MAE: mean absolute error; RMSE: root-mean-square error; SD: standard deviation; Max­(+): largest positive deviation; Max(−): largest negative deviation; R: linear correlation coefficient.

The RAS­(*h*,*p*) results exhibit a significant basis set dependence, with a clear improvement when using the larger def2-TZVP basis compared to 6-31G­(d), as reflected in various statistical indicators. Specifically, the larger basis set reduces the systematic overestimation of singlet excitation energies: the MAE decreases by 0.14 eV, the largest positive deviation is reduced by about 1 eV, and the standard deviation improves noticeably. These gains carry over to the RAS­(DK-ε) corrected energies, where, for example, the MAE decreases from 0.27 eV with 6-31G­(d) to 0.18 eV with def2-TZVP, and the largest positive deviation is reduced by nearly half.

### 4.6 Low-Lying Excitations in Heptazine

As a final test of the RASCI(2) methodology, we examine the lowest singlet and triplet excited states of heptazine (1,3,4,6,7,9,9b-heptaazaphenalene, or tri-s-triazine), a larger and electronically more complex system than those studied in the previous sections.

Heptazine, like other triangular nitrogen-rich organic compounds,^73^ exhibits a distinctive electronic structure that challenges conventional rules. In particular, it violates Hund’s rule, leading to an inversion of the lowest singlet and triplet energy ordering, as previously demonstrated.^74^ This near-degeneracy of the S_1_ and T_1_ states arises from a combination of its rigid, highly symmetric nuclear framework and the unusual spatial distribution of the frontier orbitals: the HOMO is localized exclusively on the six peripheral nitrogen atoms, while the LUMO resides on the six carbon atoms and the central nitrogen atom. Consequently, the HOMO → LUMO transition corresponds to an “internal” charge-transfer excitation with a near-zero exchange integral, which makes the singlet–triplet splitting extremely sensitive to electron correlation effects. Accurate prediction of this inversion therefore requires electronic structure methods capable of capturing the subtle balance between exchange interactions and dynamic correlation. In this context, heptazine represents a stringent test case for the performance of the RASCI(2) approach in reproducing both singlet and triplet excitation energies, as well as the singlet–triplet gap. Table 6 summarizes the low-lying singlet and triplet excitation energies and singlet–triplet gaps obtained using the RAS­(*h*,*p*) and RAS­(DK-ε) approaches, in comparison with results from highly correlated reference methods reported in the literature.

**Table 6:** Vertical Excitation Energies of the *S*_1_ and *T*_1_ States and the Corresponding Singlet–Triplet Gaps (in eV) of Heptazine Computed with the RAS­(*h*,*p*) and RAS­(DK-ε) Approaches Using the def2-TZVP Basis Set, Compared with Highly Correlated Reference Values from the Literature^a^

method	*E*(*S*_1_)	*E*(*T*_1_)	Δ*E*_*ST*_
RAS(*h*,*p*)/(2,2)	4.865	4.650	0.215
RAS(*h*,*p*)/(4,4)	4.322	4.179	0.143
RAS(*h*,*p*)/(6,6)	3.960	4.039	–0.079
RAS(*h*,*p*)/(8,8)	3.968	4.047	–0.079
RAS(DK-ε)/(2,2)	3.050	3.145	–0.095
RAS(DK-ε)/(4,4)	2.674	2.777	–0.103
RAS(DK-ε)/(6,6)	2.546	2.770	–0.224
RAS(DK-ε)/(8,8)	2.594	2.808	–0.214
CASPT2^b^	2.326	2.551	–0.225
SC-NEVPT2^c^	3.259	3.398	–0.139
RASPT2^d^	2.54	2.67	–0.13
CC3^e^	2.693	2.898	–0.205
Best^f^	2.717	2.936	–0.219

a The size of the RAS2 space (number of electrons, number of orbitals) is given in parentheses. Δ*E*_*ST*_ = *E*(*S*_1_) – *E*(*T*_1_).

b CASPT2/cc-pVDZ from ref 74.

c SC-NEVPT2/def2-TZVP from ref 75.

d RASPT2/def2-TZVP from ref 76.

e CC3/aug-cc-pVDZ from ref 77.

f Theoretical best estimate from ref 77.

Electronic transition energies to the lowest singlet and triplet states of heptazine are substantially overestimated by RAS­(*h*,*p*). Although enlarging the fully correlated space (RAS2) generally improves the results, both *S*_1_ and *T*_1_ energies remain overestimated by at least 1 eV, underscoring the limitations of the one-hole/one-particle approximation in recovering the electron correlation needed for an accurate description of these states. With the smallest RAS2 space (two electrons in the frontier orbitals), RAS­(*h*,*p*) fails to reproduce the experimentally established singlet–triplet inversion, predicting the *S*_1_ state more than 0.2 eV above *T*_1_. Increasing the size of RAS2 progressively reduces the singlet–triplet gap and ultimately restores the correct energetic ordering, *E*(*S*_1_) < *E*(*T*_1_), although the magnitude of the gap then becomes underestimated.

The application of second-order corrections through the DK partition, combined with a level shift of ε = 0.55 a.u., substantially improves the overall description. With the minimal RAS2 space, RAS­(DK-ε) still yields excitation energies that are too high, despite recovering the correct ordering. However, when larger RAS2 spaces are employed, RAS­(DK-ε) delivers significantly improved *S*_1_ and *T*_1_ energies and accurately reproduces the singlet–triplet separation, achieving values in very good agreement with high-level reference data.

These results validate the robustness of the RAS­(DK-ε) approach for describing low-lying excitations in more complex organic chromophores such as heptazine, demonstrating that its reliability extends beyond the benchmark systems analyzed in the earlier sections.

## 5 Conclusions

This work assesses the performance of second-order perturbative corrections to RAS­(*h*,*p*) transition energies for valence singlet and triplet excited states in organic molecules. Our analysis demonstrates that both EN and DK perturbation schemes can correct the systematic overestimation of excitation energies inherent to the hole-and-particle approximation, albeit with markedly different efficiencies.

The absence of effective dynamic correlation in RAS­(*h*,*p*) leads to singlet excitation energies overestimated by roughly 0.8 eV, whereas errors for triplet states are significantly smaller. RAS­(EN) provides only marginal improvement and, in fact, increases the statistical spread of the results while reducing correlation with high-level reference data. In contrast, the DK partition yields a more systematic correction, though it often overshoots, producing excitation energies that are too low. This overcorrection can be effectively mitigated by introducing a level shift, yielding highly accurate results, comparable in quality to those obtained with the well-established NEVPT2 correction to CASSCF wave functions.

For the systems studied, the optimal level shift for singlets is ε = 0.55 a.u., while triplets require a larger ε to offset the smaller RAS­(*h*,*p*) overestimation. The optimal shift may depend on the nature of the transition (e.g., ππ* vs nπ*) and molecular characteristics, but overall, a range of 0.4 ≤ ε ≤ 0.6 a.u. provides a robust compromise for valence excitations in organic molecules.

Decomposition of the second-order contributions reveals that 1*h*1*p* terms dominate the improvement, acting together with the variational 1*h* and 1*p* configurations in a way that resembles a state-specific orbital rotation. This suggests the possibility of developing computationally cheaper second-order schemes that neglect the more expensive and less impactful 1*h*2*p* and 2*h*2*p* terms.

Basis set tests show that DK-based corrections exhibit only a weak basis set dependence, especially when compared to RAS­(*h*,*p*). This feature opens the door to efficient composite strategies, in which RAS­(*h*,*p*) energies are computed with a large basis set and subsequently corrected via second order using a smaller basis, achieving significant savings with minimal accuracy loss.

Finally, the transferability of the proposed methodology has been assessed through the calculation of the lowest singlet and triplet excited states of heptazine. The resulting excitation energies, when compared with high-level reference data from the literature, highlight the ability of RAS­(DK−ε) to accurately describe electronic transitions in larger and more complex systems, where a balanced and flexible treatment of electron correlation is essential.

## Supplementary Material



## Notes

Size-intensivity test, excitation energies computed with the 6-31G­(d) basis, excitation energies without 2*h*2*p* contributions, dependence of RAS­(EN) excitation energies with the energy level shift, and a list with states included in the RASCI calculations (PDF)
