# Supplementary material for: Assessing Second-Order Perturbative Corrections to Restricted Active Space CI for Valence Excitations in Organic Molecules
Source: J Phys Chem A. 2025 Dec 6;129(50):11736–48. doi: 10.1021/acs.jpca.5c06818 (PMC12720244; doi:10.1021/acs.jpca.5c06818)
Supplement: Supplementary file 1 [file jp5c06818_si_001.pdf]

Supporting Information: Assessing Second-Order Perturbative Corrections to Restricted Active Space CI for Valence Excitations in Organic Molecules

Janaarthana Babu Perumal Marisami[†,‡] and David Casanova[*,†,¶]

†*Donostia International Physics Center (DIPC), 20018 Donostia, Euskadi, Spain*

‡*Polimero eta Material Aurreratuak: Fisika, Kimika eta Teknologia Saila, Euskal Herriko Unibertsitatea (UPV/EHU), PK 1072, 20080 Donostia, Euskadi, Spain*

E-mail: david.casanova@dipc.org

Contents

1 Size intensivity test **S3**

2 Excitation energies with 6-31G(d) **S3**

3 Comparison between 6-31G(d) and def2-TZVP results **S8**

4 Role of $2h2p$ in excitation energies **S9**

5 Dependence of Epstein–Nesbet (EN-ε) excitation energies on the energy level shift **S15**

6 States included in RASCI calculations **S16**

References S20

1 Size intensivity test

Table S1: Absolute energy difference, E(dimer)-$E(C_2H_4)$-$E(CH_2O)$ (in mHa), and excitation energy difference, ΔE(dimer)-$\Delta E(C_2H_4)$-$\Delta E(CH_2O)$ (in eV), of RAS(h,p) and RAS(DK) with the def2-TZVP basis set for ethene, formaldehyde, and a noninteracting ethene-formaldehyde supermolecule separated by 50 Å.

method	state	Abs. energy	Exc. energy
RAS(h,p)	T_1 (CH_2O)	10	-0.020
	T_1 (C_2H_4)	15	0.110
	S_1 (CH_2O)	9	-0.051
	S_1 (C_2H_4)	13	-0.325
RAS(DK)	T_1 (CH_2O)	-5	-0.106
	T_1 (C_2H_4)	2	0.100
	S_1 (CH_2O)	-5	-0.108
	S_1 (C_2H_4)	21	-0.077

2 Excitation energies with 6-31G(d)

Table S2: Vertical transition energies (in eV) to excited singlet states in computed at the RASCI(h,p), RASCI(2) with EN and DK partitions, and NEVPT2 levels with the 6-31G(d) basis set. RAS(DK-ε) refers to excitation energies obtained with a $\varepsilon = 0.55$ a.u. level shift.

molecule	state	RAS(h,p)	RAS(EN)	RAS(DK)	RAS(DK-ε)	NEVPT2	Best
ethene	$1^1B_{1u}(\pi \to \pi^*)$	10.47	7.31	8.60	9.17	8.64	7.80
butadiene	$1^1B_u(\pi \to \pi^*)$	8.32	5.29	6.54	7.08	6.21	6.18
	$2^1A_g(\pi \to \pi^*)$	6.73	7.51	6.57	6.69	6.80	6.55
hexatriene	$2^1A_g(\pi \to \pi^*)$	5.56	6.60	5.27	5.43	5.56	5.09
	$1^1B_u(\pi \to \pi^*)$	6.67	4.25	5.49	5.97	4.84	5.10
octatetraene	$2^1A_g(\pi \to \pi^*)$	4.77	6.08	4.42	4.59	4.72	4.47
	$1^1B_u(\pi \to \pi^*)$	5.90	3.55	4.86	5.29	4.04	4.66
cyclopropene	$1^1B_1(\sigma \to \pi^*)$	7.57	6.43	6.67	6.99	6.85	6.76
	$1^1B_2(\pi \to \pi^*)$	8.92	5.49	7.10	7.71	7.07	7.06

Table S2: (*Continued.*)

molecule	state	RAS(h,p)	RAS(EN)	RAS(DK)	RAS(DK-ε)	NEVPT2	Best
cyclopentadiene	$1^1B_2(\pi \rightarrow \pi^*)$	7.24	4.13	5.55	6.06	5.21	5.55
	$2^1A_1(\pi \rightarrow \pi^*)$	6.68	7.16	6.19	6.42	6.72	6.31
norbornadiene	$1^1A_2(\pi \rightarrow \pi^*)$	7.09	3.84	5.38	5.90	5.04	5.34
	$1^1B_2(\pi \rightarrow \pi^*)$	8.07	4.53	5.99	6.66	5.79	6.11
benzene	$1^1B_{2u}(\pi \rightarrow \pi^*)$	4.99	5.90	4.91	5.00	5.21	5.08
	$1^1B_{1u}(\pi \rightarrow \pi^*)$	7.84	5.91	6.55	6.92	6.40	6.54
naphthalene	$1^1B_{3u}(\pi \rightarrow \pi^*)$	4.45	4.52	3.74	4.01	4.37	4.24
	$1^1B_{2u}(\pi \rightarrow \pi^*)$	6.08	4.39	4.84	5.23	4.37	4.77
furan	$1^1B_2(\pi \rightarrow \pi^*)$	8.02	5.56	6.43	6.93	6.42	6.32
	$2^1A_1(\pi \rightarrow \pi^*)$	6.79	7.38	6.37	6.58	6.75	6.57
pyrrole	$2^1A_1(\pi \rightarrow \pi^*)$	6.58	7.13	6.21	6.39	6.56	6.37
	$1^1B_2(\pi \rightarrow \pi^*)$	7.91	6.27	6.59	7.04	6.78	6.57
imidazole	$2^1A'\,(\pi \rightarrow \pi^*)$	7.00	7.22	6.43	6.66	6.80	6.19
	1^1A" $(n \rightarrow \pi^*)$	7.25	7.11	6.47	6.75	6.97	6.81
pyridine	$1^1B_1(n \rightarrow \pi^*)$	5.54	5.09	4.63	4.93	5.26	4.59
	$1^1B_2(\pi \rightarrow \pi^*)$	5.16	5.98	5.00	5.12	5.33	4.85
	$1^1A_2(n \rightarrow \pi^*)$	6.29	5.49	5.04	5.45	5.46	5.11
	$2^1A_1(\pi \rightarrow \pi^*)$	7.80	6.42	6.66	7.00	7.09	6.26
pyrazine	$1^1B_{3u}(n \rightarrow \pi^*)$	4.64	4.34	3.94	4.17	4.20	3.95
	$1^1B_{2u}(\pi \rightarrow \pi^*)$	5.11	5.71	4.85	5.00	5.31	4.64
	$1^1A_u(n \rightarrow \pi^*)$	5.68	5.26	4.68	5.01	4.93	4.81
	$1^1B_{2g}(n \rightarrow \pi^*)$	5.98	6.10	5.33	5.57	5.86	5.56
	$1^1B_{1u}(\pi \rightarrow \pi^*)$	8.04	6.13	6.77	7.13	6.76	6.58
	$1^1B_{1g}(n \rightarrow \pi^*)$	7.29	7.33	6.24	6.62	6.77	6.60
pyrimidine	$1^1B_1(n \rightarrow \pi^*)$	5.08	4.51	4.11	4.42	4.52	4.55
	$1^1A_2(n \rightarrow \pi^*)$	5.60	4.90	4.50	4.85	4.81	4.91

Table S2: (*Continued.*)

molecule	state	RAS(h,p)	RAS(EN)	RAS(DK)	RAS(DK-ε)	NEVPT2	Best
pyridazine	$1^1B_1(n \to \pi^*)$	4.51	3.94	3.56	3.86	3.92	3.78
	$2^1A_1(\pi \to \pi^*)$	5.27	6.09	5.09	5.20	5.46	5.18
s-triazine	$1^1A_1''(n \to \pi^*)$	5.34	4.97	4.38	4.70	4.65	4.60
	$1^1A_2''(n \to \pi^*)$	5.34	4.76	4.94	5.11	4.88	4.66
	$1^1E''(n \to \pi^*)$	5.38	4.73	4.30	4.64	4.87	4.71
	$1^1A_2'(\pi \to \pi^*)$	5.77	6.68	5.57	5.69	5.92	5.79
s-tetrazine	$1^1B_{3u}(n \to \pi^*)$	3.31	1.76	1.97	2.38	2.41	2.24
	$1^1B_{2u}(\pi \to \pi^*)$	5.20	6.04	4.98	5.12	5.47	4.91
formaldehyde	$1^1A_2(n \to \pi^*)$	4.54	3.68	3.71	3.94	4.22	3.88
	$2^1A_1(\pi \to \pi^*)$	10.66	8.93	9.50	9.82	8.79	9.30
acetone	$1^1A_2(n \to \pi^*)$	4.88	4.16	3.91	4.21	4.47	4.40
	$2^1A_1(\pi \to \pi^*)$	10.56	8.22	8.82	9.37	9.28	9.40
p-benzoquinone	$1^1B_{1g}(n \to \pi^*)$	3.41	3.21	2.08	2.51	3.00	2.78
	$1^1A_u(n \to \pi^*)$	3.51	3.34	2.12	2.58	2.99	2.80
	$1^1B_{3g}(\pi \to \pi^*)$	5.23	4.26	4.17	4.53	4.35	4.25
	$1^1B_{1u}(\pi \to \pi^*)$	6.51	4.37	5.14	5.56	4.85	5.29
formamide	$1^1A''\,(n \to \pi^*)$	6.10	5.64	5.38	5.61	5.93	5.63
	$2^1A'\,(\pi \to \pi^*)$	8.56	6.89	7.05	7.56	7.58	7.44
acetamide	$1^1A''\,(n \to \pi^*)$	6.10	5.63	5.14	5.45	5.97	5.80
	$2^1A'\,(\pi \to \pi^*)$	8.66	6.60	6.78	7.40	7.48	7.27
propanamide	$1^1A''\,(n \to \pi^*)$	6.13	5.37	4.98	5.36	5.99	5.72
cytosine	$2^1A'\,(\pi \to \pi^*)$	5.32	4.73	4.12	4.52	4.70	4.66
	$1^1A''(n \to \pi^*)$	5.57	5.99	4.39	4.80	5.50	4.87
	$2^1A''(n \to \pi^*)$	6.00	6.47	4.68	5.14	5.73	5.26
	$3^1A'\,(\pi \to \pi^*)$	6.54	5.82	4.96	5.50	5.65	5.62
thymine	$1^1A''\,(n \to \pi^*)$	6.12	5.95	4.26	4.47	4.96	4.82

Table S2: (*Continued.*)

molecule	state	RAS(h,p)	RAS(EN)	RAS(DK)	RAS(DK-ε)	NEVPT2	Best
	$2^1A'\,(\pi \to \pi^*)$	6.47	4.57	4.76	5.32	5.05	5.20
	$2^1A''\,(n \to \pi^*)$	7.34	6.76	5.63	5.98	6.49	6.16
	$3^1A'\,(\pi \to \pi^*)$	7.52	6.04	5.45	6.13	6.32	6.27
uracil	$1^1A''\,(n \to \pi^*)$	5.20	5.76	4.01	4.41	4.92	4.80
	$2^1A'\,(\pi \to \pi^*)$	6.43	5.01	4.93	5.42	5.27	5.35
	$2^1A''\,(n \to \pi^*)$	6.82	6.98	5.49	5.92	6.42	6.10
	$3^1A'\,(\pi \to \pi^*)$	7.27	6.36	5.48	6.08	6.22	6.26
adenine	$1^1A''\,(n \to \pi^*)$	6.38	5.11	4.46	5.11	5.36	5.12
	$2^1A'\,(\pi \to \pi^*)$	6.00	4.92	4.50	5.01	5.43	5.25
	$3^1A'\,(\pi \to \pi^*)$	6.50	4.77	4.96	5.48	5.07	5.25
	$2^1A''\,(n \to \pi^*)$	7.10	5.67	5.22	5.85	6.07	5.75

Table S3: Vertical transition energies (in eV) to excited triplet states in computed at the RASCI(h,p), RASCI(2) with DK and EN partitions, and NEVPT2 levels with the 6-31G(d) basis set. RAS(DK-ε) refers to excitation energies obtained with a $\varepsilon = 0.55$ a.u. level shift.

molecule	state	RAS(h,p)	RAS(EN)	RAS(DK)	RAS(DK-ε)	NEVPT2	Best
ethene	$1^3B_{1u}(\pi \to \pi^*)$	4.36	4.92	4.53	4.52	4.60	4.50
butadiene	$1^3B_u(\pi \to \pi^*)$	3.30	3.84	3.27	3.32	3.38	3.20
	$1^3A_g(\pi \to \pi^*)$	5.07	5.90	5.16	5.18	5.27	5.08
hexatriene	$1^3B_u(\pi \to \pi^*)$	2.72	3.35	2.60	2.67	2.73	2.40
	$1^3A_g(\pi \to \pi^*)$	4.25	5.26	4.23	4.28	4.39	4.15
octatetraene	$1^3B_u(\pi \to \pi^*)$	2.35	3.13	2.19	2.28	2.32	2.20
	$1^3A_g(\pi \to \pi^*)$	3.64	4.82	3.57	3.63	3.72	3.55
cyclopropene	$1^3B_2(\pi \to \pi^*)$	4.37	4.73	4.27	4.34	4.54	4.34
	$1^3B_1(\sigma \to \pi^*)$	7.17	6.39	6.44	6.71	6.58	6.62

Table S3: (*Continued.*)

molecule	state	RAS(h,p)	RAS(EN)	RAS(DK)	RAS(DK-ε)	NEVPT2	Best
cyclopentadiene	$1^3B_1(\pi \rightarrow \pi^*)$	3.21	3.71	3.12	3.19	3.32	3.25
	$1^3A_1(\pi \rightarrow \pi^*)$	4.98	5.80	4.90	4.98	5.22	5.09
norbornadiene	$1^3A_2(\pi \rightarrow \pi^*)$	3.78	4.20	3.38	3.56	3.79	3.72
	$1^3B_2(\pi \rightarrow \pi^*)$	4.18	5.06	3.83	4.00	4.30	4.16
benzene	$1^3B_{1u}(\pi \rightarrow \pi^*)$	3.88	5.02	4.05	4.05	4.32	4.15
	$1^3E_{1u}(\pi \rightarrow \pi^*)$	4.91	5.56	4.75	4.85	4.98	4.86
naphthalene	$1^3B_{2u}(\pi \rightarrow \pi^*)$	3.08	3.73	2.79	2.92	3.26	3.11
	$1^3B_{3u}(\pi \rightarrow \pi^*)$	4.34	4.49	3.74	3.96	4.24	4.18
furan	$1^3B_2(\pi \rightarrow \pi^*)$	4.05	4.82	4.09	4.13	4.33	4.17
	$1^3A_1(\pi \rightarrow \pi^*)$	5.39	6.29	5.35	5.42	5.62	5.48
pyrrole	$1^3B_2(\pi \rightarrow \pi^*)$	4.40	5.14	4.40	4.45	4.73	4.48
	$1^3A_1(\pi \rightarrow \pi^*)$	5.51	6.22	5.38	5.47	5.68	5.51
imidazole	$1^3A'\,(\pi \rightarrow \pi^*)$	4.72	5.33	4.62	4.69	4.77	4.69
	$2^3A'\,(\pi \rightarrow \pi^*)$	5.89	6.50	5.68	5.78	5.89	5.79
	1^3A" $(n \rightarrow \pi^*)$	6.72	6.71	6.02	6.27	6.46	6.37
pyridine	$1^3A_1(\pi \rightarrow \pi^*)$	4.09	5.23	4.22	4.24	4.47	4.06
	$1^3B_1(n \rightarrow \pi^*)$	4.99	4.64	4.10	4.82	4.58	4.64
	$1^3B_2(\pi \rightarrow \pi^*)$	4.96	5.39	4.70	4.39	4.58	4.25
	$2^3A_1(\pi \rightarrow \pi^*)$	5.08	5.71	4.92	5.01	5.13	4.91
s-tetrazine	$1^3B_{3u}(n \rightarrow \pi^*)$	2.67	1.25	1.31	1.72	1.64	1.89
formaldehyde	$1^3A_2(\pi \rightarrow \pi^*)$	4.07	3.38	3.36	3.55	3.75	3.50
	$1^3A_1(\pi \rightarrow \pi^*)$	5.90	6.39	5.94	5.97	6.06	5.87
acetone	$1^3A_2(n \rightarrow \pi^*)$	4.49	3.93	3.61	3.88	4.10	4.05
	$1^3A_1(\pi \rightarrow \pi^*)$	5.92	6.83	5.89	5.96	6.06	6.03
p-benzoquinone	$1^3B_{1g}(n \rightarrow \pi^*)$	3.28	3.10	1.93	2.36	2.82	2.51
	$1^3A_u(n \rightarrow \pi^*)$	3.16	2.98	1.89	2.30	2.82	2.62

Table S3: (*Continued.*)

molecule	state	RAS(h,p)	RAS(EN)	RAS(DK)	RAS(DK-ε)	NEVPT2	Best
formamide	1^3A" $(n \rightarrow \pi^*)$	5.77	5.40	5.12	5.33	5.64	5.36
	$1^3A'(\pi \rightarrow \pi^*)$	6.01	5.90	5.49	5.68	5.81	5.74
acetamide	1^3A" $(n \rightarrow \pi^*)$	5.77	5.41	4.89	5.17	5.52	5.42
	$1^3A'(n \rightarrow \pi^*)$	6.23	6.15	5.43	5.70	5.63	5.88
propanamide	1^3A" $(n \rightarrow \pi^*)$	5.79	5.22	4.74	5.08	5.54	5.45
	$1^3A'(\pi \rightarrow \pi^*)$	6.41	5.99	5.29	5.67	5.86	5.90

3 Comparison between 6-31G(d) and def2-TZVP results

Table S4: Statistical analysis of the errors for the triplet transition energies obtained with RAS(h,p) and RAS(DK-ε), and with 6-31G(d) and def2-TZVP basis sets.

	RAS(h,p)		RAS(DK-ε)	
	6-31G(d)	def2-TZVP	6-31G(d)	def2-TZVP
MSE	0.19	0.15	-0.04	-0.08
MAE	0.23	0.24	0.12	0.12
RMSE	0.32	0.33	0.15	0.14
SD	0.26	0.30	0.14	0.12
Max(+)	0.78	0.87	0.27	0.22
Max(-)	-0.27	-0.26	-0.37	-0.31
R	0.97	0.97	0.99	1.00

MSE: mean signed error; MAE: mean absolute error; RMSE: root mean square error; SD: standard deviation; Max(+): largest positive deviation; Max(-): largest negative deviation; R: linear correlation coefficient.

4 Role of $2h2p$ in excitation energies

Table S5: Statistical analysis of the errors in singlet and triplet excitation energies computed with RAS(DK-ε) and with RAS(DK-ε) excluding the $2h2p$ contributions, denoted RAS(DK-ε)*, using the def2-TZVP basis set. $\varepsilon = 0.55$ a.u. in both cases.

	singlets		triplets	
	RAS(DK-ε)	RAS(DK-ε)*	RAS(DK-ε)	RAS(DK-ε)*
MSE	0.02	-0.01	-0.08	-0.04
MAE	0.18	0.18	0.12	0.12
RMSE	0.23	0.23	0.14	0.15
SD	0.23	0.23	0.12	0.14
Max(+)	0.74	0.74	0.22	0.27
Max(-)	-0.55	-0.55	-0.31	-0.37
R	0.98	0.98	1.00	0.99

MSE: mean signed error; MAE: mean absolute error; RMSE: root mean square error; SD: standard deviation; Max(+): largest positive deviation; Max(-): largest negative deviation; R: linear correlation coefficient.

Table S6: Vertical transition energies (in eV) to excited singlet states computed with RAS(DK-ε) and with RAS(DK-ε) excluding the $2h2p$ contributions, denoted RAS(DK-ε)*, using the def2-TZVP basis set. $\varepsilon = 0.55$ a.u. in both cases.

molecule	state	RAS(DK-ε)	RAS(DK-ε)*	Best
ethene	$1^1B_{1u}(\pi \rightarrow \pi^*)$	8.41	8.15	7.80
butadiene	$1^1B_u(\pi \rightarrow \pi^*)$	6.57	6.53	6.18
	$2^1A_g(\pi \rightarrow \pi^*)$	6.63	6.56	6.55
hexatriene	$2^1A_g(\pi \rightarrow \pi^*)$	5.38	5.33	5.09
	$1^1B_u(\pi \rightarrow \pi^*)$	5.84	5.96	5.10
octatetraene	$2^1A_g(\pi \rightarrow \pi^*)$	4.52	4.48	4.47
	$1^1B_u(\pi \rightarrow \pi^*)$	5.01	4.97	4.66
cyclopropene	$1^1B_1(\sigma \rightarrow \pi^*)$	6.77	6.72	6.76
	$1^1B_2(\pi \rightarrow \pi^*)$	7.13	7.08	7.06
cyclopentadiene	$1^1B_2(\pi \rightarrow \pi^*)$	5.72	5.66	5.55

Table S6: (*Continued.*)

molecule	state	RAS(DK-ε)	RAS(DK-ε)*	Best
	$2^1A_1(\pi \rightarrow \pi^*)$	6.35	6.27	6.31
norbornadiene	$1^1A_2(\pi \rightarrow \pi^*)$	5.67	5.58	5.34
	$1^1B_2(\pi \rightarrow \pi^*)$	6.54	6.43	6.11
benzene	$1^1B_{2u}(\pi \rightarrow \pi^*)$	4.94	4.90	5.08
	$1^1B_{1u}(\pi \rightarrow \pi^*)$	6.56	6.50	6.54
naphthalene	$1^1B_{3u}(\pi \rightarrow \pi^*)$	3.99	3.95	4.24
	$1^1B_{2u}(\pi \rightarrow \pi^*)$	4.89	4.85	4.77
furan	$1^1B_2(\pi \rightarrow \pi^*)$	6.55	6.50	6.32
	$2^1A_1(\pi \rightarrow \pi^*)$	6.48	6.41	6.57
pyrrole	$2^1A_1(\pi \rightarrow \pi^*)$	6.29	6.22	6.37
	$1^1B_2(\pi \rightarrow \pi^*)$	6.64	6.58	6.57
imidazole	$2^1A' \, (\pi \rightarrow \pi^*)$	6.50	6.45	6.19
	$1^1A" \, (n \rightarrow \pi^*)$	6.59	6.58	6.81
pyridine	$1^1B_1(n \rightarrow \pi^*)$	4.91	4.91	4.59
	$1^1B_2(\pi \rightarrow \pi^*)$	5.03	5.00	4.85
	$1^1A_2(n \rightarrow \pi^*)$	5.44	5.43	5.11
	$2^1A_1(\pi \rightarrow \pi^*)$	6.70	6.66	6.26
pyrazine	$1^1B_{3u}(n \rightarrow \pi^*)$	4.08	4.08	3.95
	$1^1B_{2u}(\pi \rightarrow \pi^*)$	4.90	4.88	4.64
	$1^1A_u(n \rightarrow \pi^*)$	5.03	5.02	4.81
	$1^1B_{2g}(n \rightarrow \pi^*)$	5.45	5.44	5.56
	$1^1B_{1u}(\pi \rightarrow \pi^*)$	6.88	6.85	6.58
	$1^1B_{1g}(n \rightarrow \pi^*)$	6.54	6.53	6.60
pyrimidine	$1^1B_1(n \rightarrow \pi^*)$	4.46	4.46	4.55
	$1^1A_2(n \rightarrow \pi^*)$	4.91	4.90	4.91
pyridazine	$1^1B_1(n \rightarrow \pi^*)$	3.82	3.83	3.78

Table S6: (*Continued.*)

molecule	state	RAS(DK-ε)	RAS(DK-ε)*	Best
	$2^1A_1(\pi \to \pi^*)$	5.15	5.12	5.18
s-triazine	$1^1A_1''(n \to \pi^*)$	4.63	4.67	4.60
	$1^1A_2''(n \to \pi^*)$	4.63	4.63	4.66
	$1^1E''(n \to \pi^*)$	4.67	4.62	4.71
	$1^1A_2'(\pi \to \pi^*)$	5.62	5.60	5.79
s-tetrazine	$1^1B_{3u}(n \to \pi^*)$	2.37	2.38	2.24
	$1^1B_{2u}(\pi \to \pi^*)$	5.05	5.05	4.91
formaldehyde	$1^1A_2(n \to \pi^*)$	4.03	4.03	3.88
	$2^1A_1(\pi \to \pi^*)$	9.42	9.41	9.30
acetone	$1^1A_2(n \to \pi^*)$	4.22	4.20	4.40
	$2^1A_1(\pi \to \pi^*)$	8.85	8.86	9.40
p-benzoquinone	$1^1B_{1g}(n \to \pi^*)$	2.53	2.52	2.78
	$1^1A_u(n \to \pi^*)$	2.62	2.59	2.80
	$1^1B_{3g}(\pi \to \pi^*)$	4.29	4.30	4.25
	$1^1B_{1u}(\pi \to \pi^*)$	5.33	5.33	5.29
formamide	$1^1A''\,(n \to \pi^*)$	5.62	5.59	5.63
	$2^1A'\,(\pi \to \pi^*)$	7.38	7.35	7.44
acetamide	$1^1A''\,(n \to \pi^*)$	5.46	5.43	5.80
	$2^1A'\,(\pi \to \pi^*)$	7.20	7.18	7.27
propanamide	$1^1A''\,(n \to \pi^*)$	5.42	5.36	5.72
cytosine	$2^1A'\,(\pi \to \pi^*)$	4.45	4.44	4.66
	$1^1A''(n \to \pi^*)$	4.77	4.78	4.87
	$2^1A''(n \to \pi^*)$	5.05	5.06	5.26
	$3^1A'\,(\pi \to \pi^*)$	5.49	5.46	5.62
thymine	$1^1A''\,(n \to \pi^*)$	4.50	4.43	4.82
	$2^1A'\,(\pi \to \pi^*)$	5.12	5.11	5.20

Table S6: (*Continued.*)

molecule	state	RAS(DK-ε)	RAS(DK-ε)*	Best
	$2^1A''\,(n \to \pi^*)$	5.92	5.90	6.16
	$3^1A'\,(\pi \to \pi^*)$	6.12	6.08	6.27
uracil	$1^1A''\,(n \to \pi^*)$	4.45	4.39	4.80
	$2^1A'\,(\pi \to \pi^*)$	5.24	5.23	5.35
	$2^1A''\,(n \to \pi^*)$	5.92	5.88	6.10
	$3^1A'\,(\pi \to \pi^*)$	6.15	6.10	6.26
adenine	$1^1A''\,(n \to \pi^*)$	5.09	5.08	5.12
	$2^1A'\,(\pi \to \pi^*)$	5.01	4.98	5.25
	$3^1A'\,(\pi \to \pi^*)$	5.33	5.30	5.25
	$2^1A''\,(n \to \pi^*)$	5.77	5.77	5.75

Table S7: Vertical transition energies (in eV) to excited triplet states computed with RAS(DK-ε) and with RAS(DK-ε) excluding the $2h2p$ contributions, denoted RAS(DK-ε)*, using the def2-TZVP basis set. $\varepsilon = 0.55$ a.u. in both cases.

molecule	state	RAS(DK-ε)	RAS(DK-ε)*	Best
ethene	$1^3B_{1u}(\pi \to \pi^*)$	4.49	4.44	4.50
butadiene	$1^3B_u(\pi \to \pi^*)$	3.27	3.23	3.20
	$1^3A_g(\pi \to \pi^*)$	5.11	5.05	5.08
hexatriene	$1^3B_u(\pi \to \pi^*)$	2.62	2.58	2.40
	$1^3A_g(\pi \to \pi^*)$	4.25	4.20	4.15
octatetraene	$1^3B_u(\pi \to \pi^*)$	2.18	2.15	2.20
	$1^3A_g(\pi \to \pi^*)$	3.57	3.51	3.55
cyclopropene	$1^3B_2(\pi \to \pi^*)$	4.33	4.29	4.34
	$1^3B_1(\sigma \to \pi^*)$	6.45	6.40	6.62
cyclopentadiene	$1^3B_1(\pi \to \pi^*)$	3.21	3.15	3.25

Table S7: (*Continued.*)

molecule	state	RAS(DK-ε)	RAS(DK-ε)*	Best
	$1^3A_1(\pi \to \pi^*)$	4.97	4.90	5.09
norbornadiene	$1^3A_2(\pi \to \pi^*)$	3.56	3.48	3.72
	$1^3B_2(\pi \to \pi^*)$	3.96	3.84	4.16
benzene	$1^3B_{1u}(\pi \to \pi^*)$	4.04	3.99	4.15
	$1^3E_{1u}(\pi \to \pi^*)$	4.74	4.69	4.86
naphthalene	$1^3B_{2u}(\pi \to \pi^*)$	2.92	2.88	3.11
	$1^3B_{3u}(\pi \to \pi^*)$	3.89	3.84	4.18
furan	$1^3B_2(\pi \to \pi^*)$	4.07	4.04	4.17
	$1^3A_1(\pi \to \pi^*)$	5.37	5.30	5.48
pyrrole	$1^3B_2(\pi \to \pi^*)$	4.42	4.37	4.48
	$1^3A_1(\pi \to \pi^*)$	5.40	5.33	5.51
imidazole	$1^3A'\,(\pi \to \pi^*)$	4.66	4.63	4.69
	$2^3A'\,(\pi \to \pi^*)$	5.70	5.66	5.79
	$1^3A"\,(n \to \pi^*)$	6.17	6.15	6.37
pyridine	$1^3A_1(\pi \to \pi^*)$	4.21	4.17	4.06
	$1^3B_1(n \to \pi^*)$	4.41	4.65	4.25
	$1^3B_2(\pi \to \pi^*)$	4.69	4.40	4.64
	$2^3A_1(\pi \to \pi^*)$	4.92	4.87	4.91
s-tetrazine	$1^3B_{3u}(n \to \pi^*)$	1.77	1.78	1.89
formaldehyde	$1^3A_2(\pi \to \pi^*)$	3.47	3.61	3.50
	$1^3A_1(\pi \to \pi^*)$	5.87	5.95	5.87
acetone	$1^3A_2(n \to \pi^*)$	3.89	3.87	4.05
	$1^3A_1(\pi \to \pi^*)$	5.81	5.80	6.03
p-benzoquinone	$1^3B_{1g}(n \to \pi^*)$	2.32	2.31	2.51
	$1^3A_u(n \to \pi^*)$	2.41	2.38	2.62
formamide	$1^3A"\,(n \to \pi^*)$	5.32	5.29	5.36

Table S7: (*Continued.*)

molecule	state	RAS(DK-ε)	RAS(DK-ε)*	Best
	$1^3A'(\pi \rightarrow \pi^*)$	5.69	5.64	5.74
acetamide	1^3A" $(n \rightarrow \pi^*)$	5.20	5.16	5.42
	$1^3A'(n \rightarrow \pi^*)$	5.73	5.69	5.88
propanamide	1^3A" $(n \rightarrow \pi^*)$	5.14	5.07	5.45
	$1^3A'(\pi \rightarrow \pi^*)$	5.74	5.69	5.90

5 Dependence of Epstein–Nesbet (EN-ε) excitation energies on the energy level shift

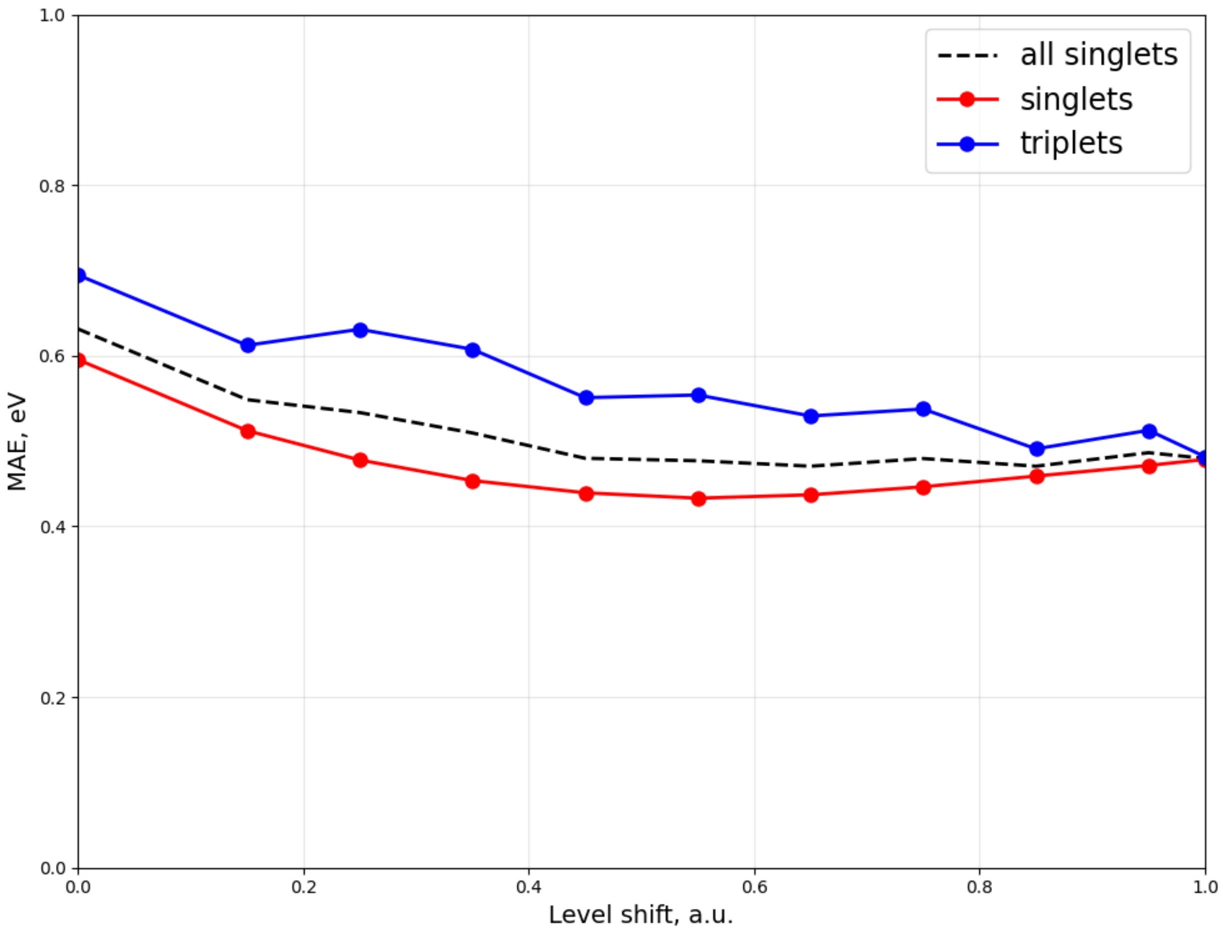

Figure S1: Dependence of the mean absolute error (MAE, in eV) on the level-shift parameter (ε, in a.u.) for RASCI(2) with the EN partition: all excitations (dashed black line), triplet states (blue), and singlet states (red).

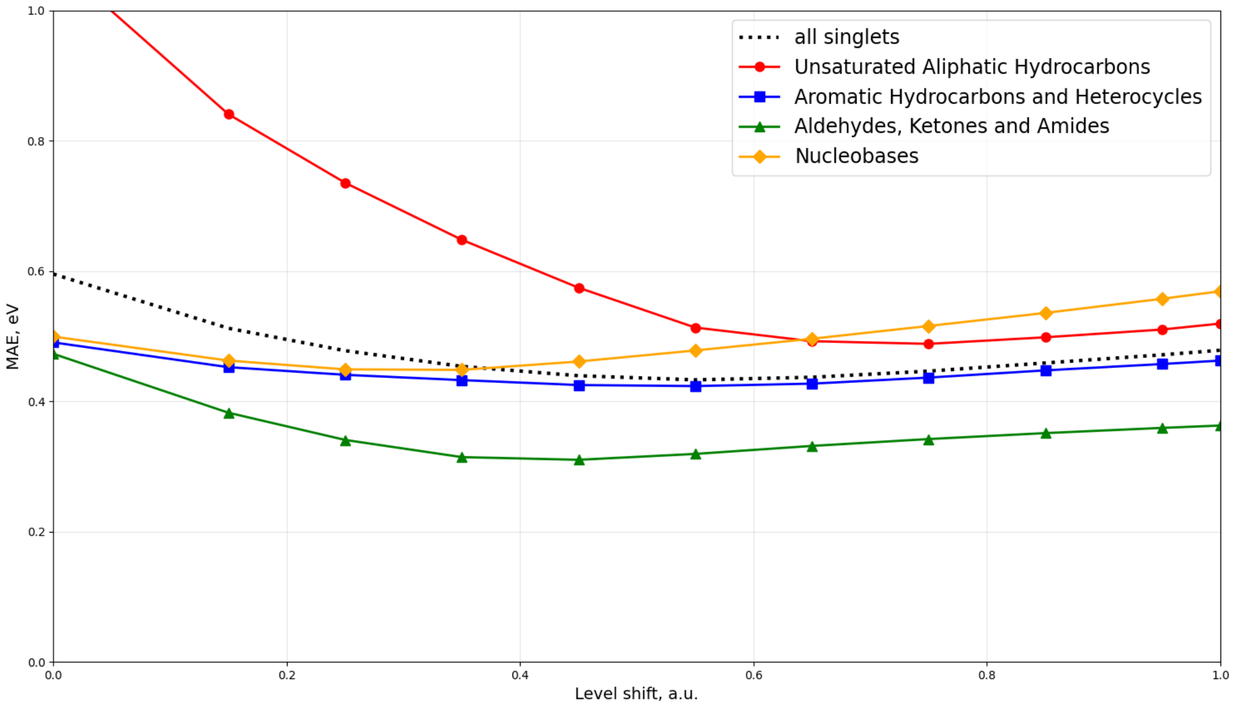

Figure S2: Dependence of the mean absolute error (MAE, in eV) on the level-shift parameter (ε, in a.u.) for RASCI(2) with the DK partition with all singlets (dashed black line) and singlet excitations grouped by different molecular families.

6 States included in RASCI calculations

Table S8: RAS2 space and electronic states included in each RASCI and RASCI(2) calculation. All symmetries were defined in the standard orientation, and the states taken into account in the RASCI computations used symmetry up to D_{2h} and its subgroups. **Bolded molecules were computed using different RAS2 space than the active space employed in reference.**[S1]

Molecule	RAS2	Irrep	Count
ethene (D_{2h})	(2,2)	A_g	1
		B_{1u}	1
E-butadiene (C_{2h})	(4,4)	B_g	2
		A_u	2
all-*E*-hexatriene (C_{2h})	(6,6)	A_g	3

Continued on next page

Table S8 – continued from previous page

Molecule	RAS2	Irrep	Count
		B_g	3
all-*E*-octatetraene (C_{2h})	(8,8)	A_u	5
		B_g	3
cyclopropene (C_{2v})	(4,3)	A_1	1
		B_1	1
		B_2	1
cyclopentadiene (C_{2v})	(4,4)	A_2	2
		B_1	2
norbornadiene (C_{2v})	(4,4)	A_1	1
		A_2	1
		B_2	1
		B_1	1
benzene (D_{2h})	(6,6)	A_u	1
		B_{1g}	1
		B_{3u}	2
		B_{2u}	2
naphthalene (D_{2h})	(10,8)	A_u	1
		B_{1g}	2
		B_{3u}	3
		B_{2u}	2
furan (C_{2v})	(6,5)	A_2	2
		B_1	3
pyrrole (C_{2v})	(6,5)	A_2	2
		B_1	3
imidazole (C_s)	(8,7)	A'	2

Continued on next page

Table S8 – continued from previous page

Molecule	RAS2	Irrep	Count
		A″	6
pyridine (C_{2v})	(8,7)	A_1	1
		A_2	2
		B_1	4
pyrazine (D_{2h})	(10,8)	A_g	1
		B_{1g}	1
		B_{2g}	1
		B_{3g}	2
		A_u	1
		B_{2u}	1
		B_{3u}	2
pyrimidine (C_{2v})	(10,8)	A_1	2
		A_2	2
		B_1	4
		B_2	1
pyridazine (C_{2v})	(10,8)	A_1	1
		A_2	3
		B_1	3
		B_1	1
s-triazine (C_{2v})	(12,9)	A_1	2
		A_2	2
		B_1	4
		B_2	1
s-tetrazine (D_{2h})	(12,10)	A_u	1
		B_{1g}	2
		B_{2g}	2

Continued on next page

Table S8 – continued from previous page

**Molecule**	**RAS2**	**Irrep**	**Count**
		B_{3g}	1
		B_{2u}	1
		B_{3u}	1
formaldehyde (C_{2v})	(6,4)	B_1	2
		B_2	1
		A_1	1
acetone (C_{2v})	(6,5)	A_1	1
		B_2	1
		B_1	3
***p*-benzoquinone** (D_{2h})	(10,8)	A_g	3
		B_{1g}	1
		B_{2g}	2
		B_{3g}	1
		A_u	1
		B_{2u}	1
		B_{3u}	2
formamide (C_s)	(6,4)	A'	1
		A''	3
**acetamide** (C_s)	(6,**5**)	A'	1
		A''	4
**propanamide** (C_s)	(6,**5**)	A'	1
		A''	4
**cytosine** (C_s)	(10,8)	A'	2
		A''	7
**thymine** (C_s)	(10,8)	A'	2

Continued on next page

Table S8 – continued from previous page

Molecule	RAS2	Irrep	Count
		A″	6
**uracil** (C_s)	(10,8)	A′	2
		A″	6
**adenine** (C_s)	(10,8)	A′	2
		A″	6

References

(S1) Schreiber, M.; Silva-Junior, M. R.; Sauer, S.; Thiel, W. Benchmarks for electronically excited states: CASPT2, CC2, CCSD, and CC3. *J. Chem. Phys.* **2008**, *128*, 134110.
